# Role of Niobium and Tantalum on the Diffusivity of Point Defects in the Passive Film of TiZrNbTa Multi-Principal Element Alloys in Sodium Chloride Solution

**DOI:** 10.3390/ma19214720

**Published:** 2026-11-06

**Authors:** Zening Wang, Jinping Wu, Yi Zhang, Yu Yan, Lijie Qiao

**Affiliations:** Beijing Advanced Innovation Center for Materials Genome Engineering, Corrosion and Protection Center, Institute for Advanced Materials and Technology, University of Science and Technology Beijing, Beijing 100083, China; wangzeningning@163.com (Z.W.);

**Keywords:** multi-principal element alloys, passive film, point defects, XPS, PDM

## Abstract

In this work, the alloying contents of Nb and Ta in TiZrNbTa refractory multi-principal element alloys (RMPEAs) were systematically tailored. Combining multiple electrochemical techniques with X-ray photoelectron spectroscopy (XPS), the passivation behavior and point defect diffusion within the passive film were quantitatively resolved and interpreted within the framework of the point defect model (PDM). The results show that all three alloys exhibit typical self-passivation behavior, with pitting potentials (*E*_pit_) exceeding 3.0 V_SCE_. As the Nb and Ta contents increase, the corrosion rate of the alloys decreases, and their resistance to metastable pitting is enhanced. The passive films exhibit n-type semiconductive characteristics, with their outer layers dominated by high-valent oxides of the constituent elements. Based on the analysis using defect chemistry and the solute–vacancy interaction model (SVIM), the doping of Nb and Ta reduces the vacancy concentration within the film while suppressing the outward diffusion of Ti cations, resulting in pronounced Ti depletion and significant Nb and Ta enrichment in the film. In addition, the incorporation of Nb and Ta weakens chloride ion adsorption and retards point defect diffusion, thereby delaying film dissolution and localized breakdown. On the other hand, Ti and Zr facilitate the rapid growth of passive films and act synergistically with Nb and Ta, thereby conferring the alloys with superior pitting resistance.

## 1. Introduction

High-entropy alloys (HEAs), also known as multi-principal element alloys (MPEAs), exhibit outstanding mechanical properties, including exceptionally high strength, good ductility, and remarkable corrosion resistance, making them highly promising for advanced applications in aerospace engineering, energy systems, cryogenic environments, and biomedical fields. Traditional biomedical implants have been predominantly manufactured using highly corrosion-resistant passivable alloys, such as Co-28Cr-6Mo, Ti-6Al-4V, and 316L stainless steel (SS). However, upon long-term implantation in the body, these alloys tend to release toxic metal ions including Co, Ni, Al, and V, which may trigger inflammatory responses [1,2]. In recent years, refractory multi-principal element alloys (RMPEAs) composed of biocompatible elements such as Ti, Zr, Hf, Nb, Ta, W, V and Mo have emerged as a research hotspot for next-generation biomedical implant materials, owing to their excellent corrosion resistance [3], superior mechanical properties [4], favorable biocompatibility [5], and tunable passive film characteristics [6]. RMPEAs comprising these elements tend to readily develop a single-phase, stable solid-solution structure, thereby inhibiting, to some extent, the onset of selective localized corrosion [7,8,9]. Furthermore, these elements themselves possess exceptionally strong oxygen affinity, which facilitates the formation of a dense and chemically stable passive film on the alloy surface. This passive layer effectively impedes the attack of aggressive ions, thereby endowing the alloys with exceptional pitting corrosion resistance [9].

The excellent corrosion resistance of RMPEAs is closely associated with the formation of a passive film, which is a thin layer composed of metal oxides or hydroxides. Unlike traditional corrosion-resistant alloys, multiple elements in the passive film of HEAs simultaneously play dominant roles, rendering the composition and structure of the passive film more complex. To date, limited studies have focused on the passivation behavior or passive film of RMPEAs. Zhou et al. [10] reported that Hf_0.5_Nb_0.5_Ta_0.5_Ti_1.5_Zr HEA exhibits excellent corrosion resistance in 3.5 wt% NaCl, with corrosion current density one-fifth that of 316L SS and pitting potential (*E*_pit_) reaching +8.36 V_SCE_. This performance stems from the uniform distribution of passivating elements in the single-phase solid solution and synergistic stabilization of the passive film by metallic Ta and OH^−^ species. The VNbTa multi-principal element alloy (MPEA) exhibits significantly superior corrosion resistance to 316L SS in 3.5 wt% NaCl solution, with an *E*_pit_ exceeding 2.0 V_SCE_ [11]. The outstanding corrosion resistance stems from the synergistic effect of the high ε_M-M_ bond strength among constituent elements, which suppresses dissolution during the initial corrosion stage, and the subsequent stabilization of the passive film by high-valence stable oxides. Wang et al. [9] reported that the Ti_46.1_Zr_23.1_Nb_15.4_V_15.4_ HEA exhibits a remarkably high *E*_pit_ of 9.907 V_SCE_ in 3.5 wt% NaCl solution, arising from a dual synergistic mechanism in which the Nb/V-doped Ti-enriched passive film reduces donor density and remains amorphous to inhibit pitting initiation, while enhanced metal–metal bonds such as Nb-V lower the dissolution current and suppress the transition from metastable to stable pits. Li et al. [12] demonstrated that the Ti_45_V_30_Zr_12_Nb_12_Al_1_ MPEA achieves superior corrosion resistance compared to the conventional Ti80 by forming a unique passive film with amorphous and bipolar semiconducting characteristics, in which Nb plays a key role by effectively blocking Cl^−^ penetration and cation dissolution. Thus, the superior corrosion resistance of RMPEAs intrinsically arises from the synergistic effects among multiple principal elements on both active dissolution and passivation behaviors. On the one hand, stronger interatomic metal–metal bonds that formed among these principal elements reduce the matrix dissolution rate at the initial corrosion stage and restrain the transition from metastable to stable pitting [13,14]. On the other hand, the synergistic action of multi-principal element cations modulates the defect concentration [15], chemical composition, microstructure [16], and semiconductor properties [17] within the passive film, suppressing anion ingress and cation dissolution and further retarding pitting nucleation processes.

According to the PDM, the growth and breakdown behavior of passive films on alloy surfaces are governed by the concentration distribution and diffusion kinetics of point defects within the film. The generation and transport mechanisms of these defects are intrinsically correlated with the alloy chemical composition. Owing to their broad composition design space, the MPEAs enable effective modulation of the chemical state and structural characteristics of the passive film, thereby affording controllable tuning of the type, density, and migration rate of internal defects. Dai et al. [18] demonstrated that Mo addition lowers the defect density and raises the resistance of the passive film on CoCrFeNi HEAs owing to the Mo enrichment in the outer film layer that promotes the formation of a more dense Cr_2_O_3_-rich inner layer. Increasing the Nb content in TiHfZrNb HEAs promotes passive film thickening, suppresses anodic dissolution, and accelerates passivation kinetics. The enrichment of Nb oxides enhances film compactness and reduces both the density and mobility of point defects, thereby inhibiting pit initiation [19]. Chen et al. [20] demonstrated that FeCoNiCrAl HEA coatings with high Cr and low Al alloying significantly reduce the defect density in the passive film and effectively inhibit both pitting and uniform corrosion. The passive film exhibits p-n heterojunction semiconductor characteristics, which impede the migration of corrosive species. Gu et al. [21] found that Al addition promotes the formation of a thicker passive film on CoCrFeNi alloys, with the film composition shifting predominantly to aluminum oxides/hydroxides. This addition also induces a transition in the semiconducting behavior from p-type to n-type, albeit accompanied by a higher defect density in the passive film. In fluoride-containing environments, the addition of Ta promotes the enrichment of Ta_2_O_5_ on the surface of TiZrHfNb MPEAs, reduces the donor defect concentration in the passive film, and enables self-repair of the passive film, thereby significantly enhancing the fluoride corrosion resistance of TiZrHfNbTa MPEAs [22]. In 0.6 M NaCl solution, the barrier layer growth kinetics of the passive film on AlTiVCr MPEAs conform to the PDM, with TiO_2_ dominating the film growth. The enrichment of TiO_2_ in the barrier layer reduces the electric field strength within the film and increases the diffusion coefficient of $V_{o}^{\cdot \cdot }$, thereby accelerating barrier layer growth even during the transpassive dissolution of V and Cr [23]. Chen et al. [24] demonstrated that CoCrFeMnNi HEAs form a bilayer amorphous multi-oxide passive film with a high-resistance barrier layer and a low-resistance outer layer. The barrier layer thickens logarithmically with time and linearly with potential, following the PDM. Elemental enrichment and depletion vary across sublayers and transition regions, regulated by both thermodynamics and kinetics. It is evident that current research on the passive films of MPEAs has been largely devoted to elucidating the effects of constituent elements on the types and population of point defects within the film. However, the kinetic behavior of defect diffusion and its linkage to the microchemical evolution of the passive film have yet to be systematically addressed. This knowledge gap has hindered a comprehensive understanding of both the growth kinetics of the passive film and the microscopic failure mechanisms triggered by corrosive species. Nonetheless, previous studies have established that the passive film growth on MPEAs conforms to the PDM, which lays the groundwork for probing the generation, migration, and annihilation of defects and for developing quantitative “composition–defect–performance” correlations.

The TiZrNbTa RMPEAs exhibit a single solid-solution phase with remarkable microstructural stability [25,26]. Coupled with their superior mechanical performance [26], corrosion resistance [27,28], and wear resistance [27], these alloys are considered to be promising candidates for next-generation biomedical implant applications. Currently, there remain substantial and crucial knowledge gaps regarding the passivation behavior and corrosion resistance mechanisms of TiZrNbTa RMPEAs, seriously impeding the optimization of their passivation performance and their practical deployment in biomedical implant applications. In the TiZrTaNb system, the Group IV elements (Ti and Zr) and Group V elements (Nb and Ta) exhibit significant differences in physicochemical properties [29,30,31,32]. However, systematic investigations into the effects of Nb and Ta contents on the pitting resistance, defect evolution, and diffusion kinetics of passive films formed on TiZrNbTa RMPEAs remain scarce, and the mechanism governing the relationship between composition and corrosion resistance remains poorly understood. To address this gap, this work systematically tunes the ratios of these two groups of elements to explore the intrinsic correlations among alloy microstructure, passive film chemical composition, corrosion resistance, and point defect diffusion behavior. This study aims to provide experimental and theoretical foundations for the compositional design of RMPEAs.

## 2. Materials and Methods

### 2.1. Material Preparation

In this study, RMPEAs were prepared from commercial purity Ti, Nb, Ta, and Zr (all with purity of 99.9 wt.%, Beijing General Research Institute for Nonferrous Metals, Beijing, China) by electric arc melting under an argon atmosphere in a water-cooled copper crucible. To ensure chemical homogeneity, the molten material was repeatedly melted five times and subsequently cast into a copper mold, ultimately yielding a homogenized alloy ingot. This compositional homogenization was achieved solely through repeated arc remelting, without any post-casting homogenization heat treatment. All characterizations in this work were performed on these as-cast ingot specimens. Each ingot was machined by wire cutting into square sheets with dimensions of 10 mm × 10 mm × 1 mm, and the surfaces were subsequently ground using #1000, #2000, #3000, and #5000 SiC sandpapers (Yingkou Sanzheng New Technology Industry Co., Ltd., Yingkou, China) in sequence.

### 2.2. Characterization of Alloy Microstructure and Passive Film Composition

The phase constitution of the alloys was determined by X-ray diffraction (XRD, D8 Advance, Bruker, Karlsruhe, Germany) using Cu-Kα radiation. XRD patterns were collected over a 2*θ* range at a scanning speed of 5°·min^−1^. The microstructure and elemental distribution of the alloys were characterized using a scanning electron microscope (SEM, Zeiss Supra 55, Carl Zeiss AG, Oberkochen, Germany) equipped with an energy-dispersive X-ray spectroscopy (EDS) system, on specimens that had been vibration-polished with a 0.04 μm SiO_2_ suspension (SiS001-JP, Truer, Suzhou, China). The chemical composition of the passive films formed on the alloy surfaces was analyzed by X-ray photoelectron spectroscopy (XPS, PHI Quantera SXM, Physical Electronics, Chanhassen, MN, USA) using monochromatized Al-Kα radiation (h_ν_ = 1486.6 eV) as the excitation source. For all XPS analyses, the photoelectron take-off angle was set at 45° and the analysis spot size was 100 μm. Survey spectra were acquired over a binding energy range of 1200 eV with a pass energy of 55 eV and a spectral resolution of 0.1 eV.

### 2.3. Electrochemical Measurements

Electrochemical tests were conducted in a conventional three-electrode electrochemical cell. A saturated calomel electrode (SCE, Model 232, Shanghai INESA Scientific Instrument Co., Ltd., Shanghai, China) was used as the reference electrode, and a platinum sheet (Wuhan Corrtest Instruments Corp., Ltd., Wuhan, China) served as the counter electrode. The effective exposed area of each working electrode was fixed at 1.0 cm^2^, with the remaining surfaces fully encapsulated by insulating epoxy resin (Shanghai Meixin Resin Co., Ltd., Shanghai, China) to eliminate stray current interference. All electrochemical measurements were performed using an Ivium Vertex electrochemical workstation (Ivium Technologies B.V., Eindhoven, The Netherlands) in a 0.9 wt.% NaCl solution (sodium chloride, Sinopharm Chemical Reagent Co., Ltd., Shanghai, China) at room temperature (25 ± 2 °C) under atmospheric pressure. The samples were mounted with copper wire (Aladdin, Shanghai, China) connections in epoxy resin, ground, degreased with alcohol (anhydrous ethanol, Sinopharm Chemical Reagent Co., Ltd., Shanghai, China), cleaned, and dried in warm air. Prior to testing, the working electrode was cathodically prepolarized at −1.2 V_SCE_ for 600 s in the electrolyte to remove the spontaneously formed air-formed oxide film on the surface [24,33]. The open-circuit potential (OCP) was recorded over a period of 12 h to ensure that the potential attained a stable value. A potentiostatic polarization (PSP) method was used for the detection of the quasi-steady-state current density (*i*_ss_) and metastable pitting current transients, which involved polarization of the sample at selected potentials for 1.5 h. Electrochemical impedance spectroscopy (EIS) was employed to determine certain properties of the oxide film, including effective capacitance, thickness, and electric field strength. The EIS measurements were conducted over a frequency range from 10^4^ Hz to 10^−2^ Hz with a peak-to-peak sinusoidal voltage signal of 10 mV. To determine the semiconducting properties of the oxides, capacitance measurements were performed by sweeping the potential from the determined anodic value to the cathodic direction at a fixed frequency of 1000 Hz using a 10 mV AC (alternating current) signal and a step rate of 25 mV·s^−1^. PSP was employed to form passive films on the specimen surfaces at potentials of 0, 0.5, 1.0, 2.0, and 3.0 V_SCE_ within the passive region identified by potentiodynamic polarization (PDP) testing. Following PSP treatment, EIS and Mott–Schottky (MS) measurements were conducted to evaluate the relevant performance parameters of the oxide films. In addition, a frequency of 18 Hz and an AC disturbance signal of 10 mV were used in the potential of zero charge (PZC) measurements.

## 3. Results

### 3.1. Phase Composition and Microstructure

**Figure 1:**
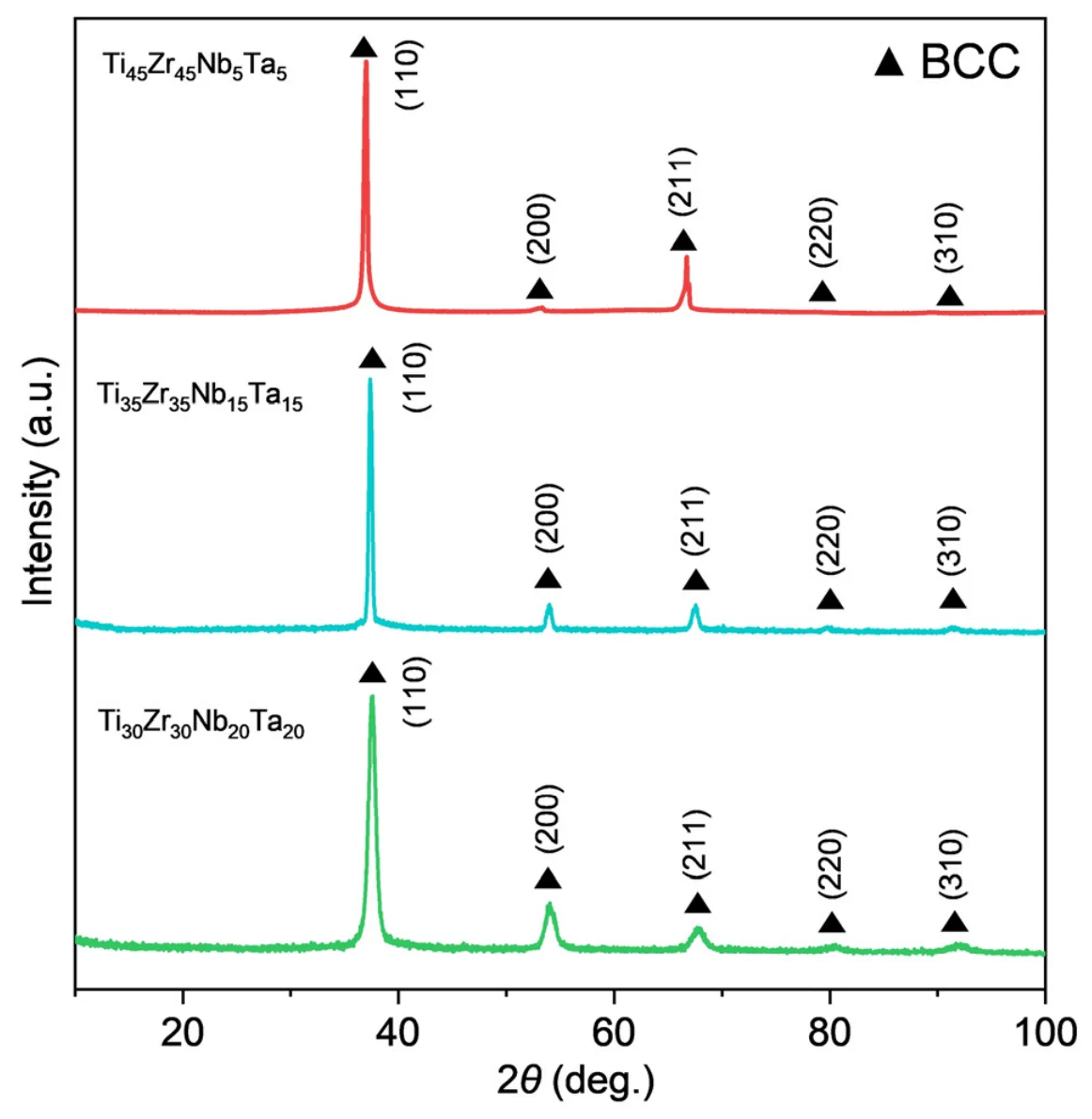
XRD patterns of all investigated samples.

Figure 1 shows the XRD patterns of the as-cast Ti_45_Zr_45_Nb_5_Ta_5_, Ti_35_Zr_35_Nb_15_Ta_15_, and Ti_30_Zr_30_Nb_20_Ta_20_ alloys. The diffraction peak positions were carefully indexed to the (110), (200), (211), (220), and (310) planes of the body-centered cubic (BCC) structure, which agrees with the reported structures of TiZrNbTa RMPEAs in the literature [34,35]. Quantitative analysis via Gaussian peak fitting yielded lattice constants of a = 3.435 ± 0.005 Å, 3.396 ± 0.002 Å, and 3.384 ± 0.004 Å for the Ti_45_Zr_45_Nb_5_Ta_5_, Ti_35_Zr_35_Nb_15_Ta_15_, and Ti_30_Zr_30_Nb_20_Ta_20_ alloys, respectively. As the combined content of Nb and Ta increases from 10 at.% to 40 at.%, the positions of the above peaks systematically shift towards higher Bragg angles, for instance, the (110) reflection moves from 36.96° to 37.56°, indicating a progressive decrease in the lattice parameters of the TiZrNbTa RMPEAs. This change in peak position is caused by the lattice contraction associated with the reduced Zr content (largest atomic radius) and the increased fractions of smaller Nb/Ta atoms [4,36]. No other diffraction peaks from intermetallic compounds or precipitates were found in the XRD patterns for these three TiZrNbTa RMPEAs. All three alloys exhibited a single BCC solid solution structure with no formation of a second phase, confirming the high phase stability of the TiZrNbTa system across the investigated composition range.

**Figure 2:**
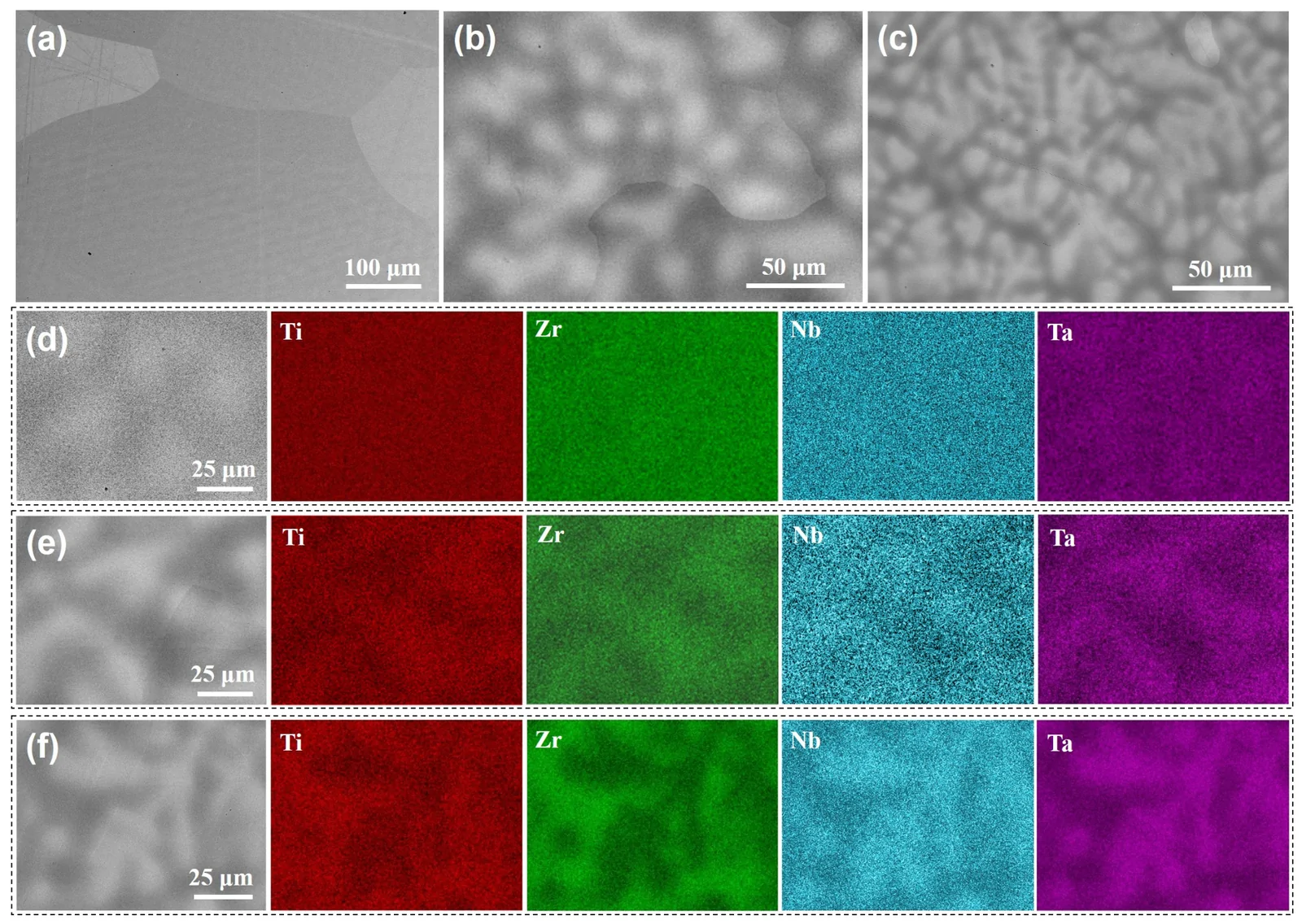
SEM back-scattered electron (BSE) images of the as-cast TiZrNbTa RMPEAs and their corresponding elemental mapping: (**a**) BSE image of the Ti_45_Zr_45_Nb_5_Ta_5_ alloy; (**b**) BSE image of the Ti_35_Zr_35_Nb_15_Ta_15_ alloy; (**c**) BSE image of the Ti_30_Zr_30_Nb_20_Ta_20_ alloy; (**d**) EDS elemental mapping of the Ti_45_Zr_45_Nb_5_Ta_5_ alloy; (**e**) EDS elemental mapping of the Ti_35_Zr_35_Nb_15_Ta_15_ alloy; (**f**) EDS elemental mapping of the Ti_30_Zr_30_Nb_20_Ta_20_ alloy.

The microstructures of the as-cast TiZrNbTa RMPEAs are presented in Figure 2a–c. Typical cast dendritic structures were observed in all three investigated alloys. Well-defined grain and grain boundary morphologies were also visible, and the grain size decreased with increasing Nb and Ta contents. The Ti_45_Zr_45_Nb_5_Ta_5_ alloy exhibited a regularly aligned fish-bone dendritic structure (Figure 2a), whereas both Ti_35_Zr_35_Nb_15_Ta_15_ and Ti_30_Zr_30_Nb_20_Ta_20_ alloys displayed typical tree-like dendritic morphologies (Figure 2b,c). The progressive refinement of grains and dendritic arms with increasing Nb and Ta alloying can be attributed to the strong grain-growth pinning effect arising from the sluggish diffusion of Nb and Ta [6,37,38]. The dendrites with light-gray contrast and interdendritic regions with dark-gray contrast reflect the redistribution of constituent elements during solidification. As shown in Figure 2d–f and Table 1, EDS mapping combined with quantitative analysis revealed that the dendritic regions were enriched in Nb and Ta, whereas Zr was markedly enriched in the interdendritic regions. Ti exhibited only a weak tendency toward interdendritic enrichment, with substantially smaller composition differences than those of Zr, Nb, and Ta. Specifically, this elemental segregation was weak in the Ti_45_Zr_45_Nb_5_Ta_5_ alloy, but pronounced segregation was observed in the other two alloys. The pronounced segregation at higher Nb and Ta contents can be attributed to enhanced solute redistribution associated with the wider solidification interval. Nb and Ta preferentially partition into the primary dendritic solid owing to their higher melting points, whereas Zr tends to remain in the residual liquid [39,40]. This difference is further supported by the mixing enthalpies: Ti–Nb (2 kJ·mol^−1^) and Ti–Ta (1 kJ·mol^−1^) are lower than Zr–Nb (4 kJ·mol^−1^) and Zr–Ta (3 kJ·mol^−1^), respectively [41], indicating that Ti is less strongly rejected from the Nb/Ta-enriched dendrites than Zr. Together with the atomic-size mismatch between Zr and Nb/Ta, this promotes Zr retention in the residual liquid and its enrichment in the interdendritic regions [42]. Thus, the pronounced dendritic contrast mainly reflects the redistribution of Nb, Ta, and Zr and does not require a comparably large Ti composition difference. Notably, despite the highest degree of solute redistribution occurring in Ti_30_Zr_30_Nb_20_Ta_20_ alloy, the XRD results confirmed that all three alloys maintained a single BCC solid-solution structure without the formation of intermetallic compounds or secondary phases, indicating that the elemental segregation within this composition range induces only localized lattice distortion without disrupting the crystallographic symmetry of the matrix.

### 3.2. Open-Circuit Potential and Potentiodynamic Polarization

**Figure 3:**
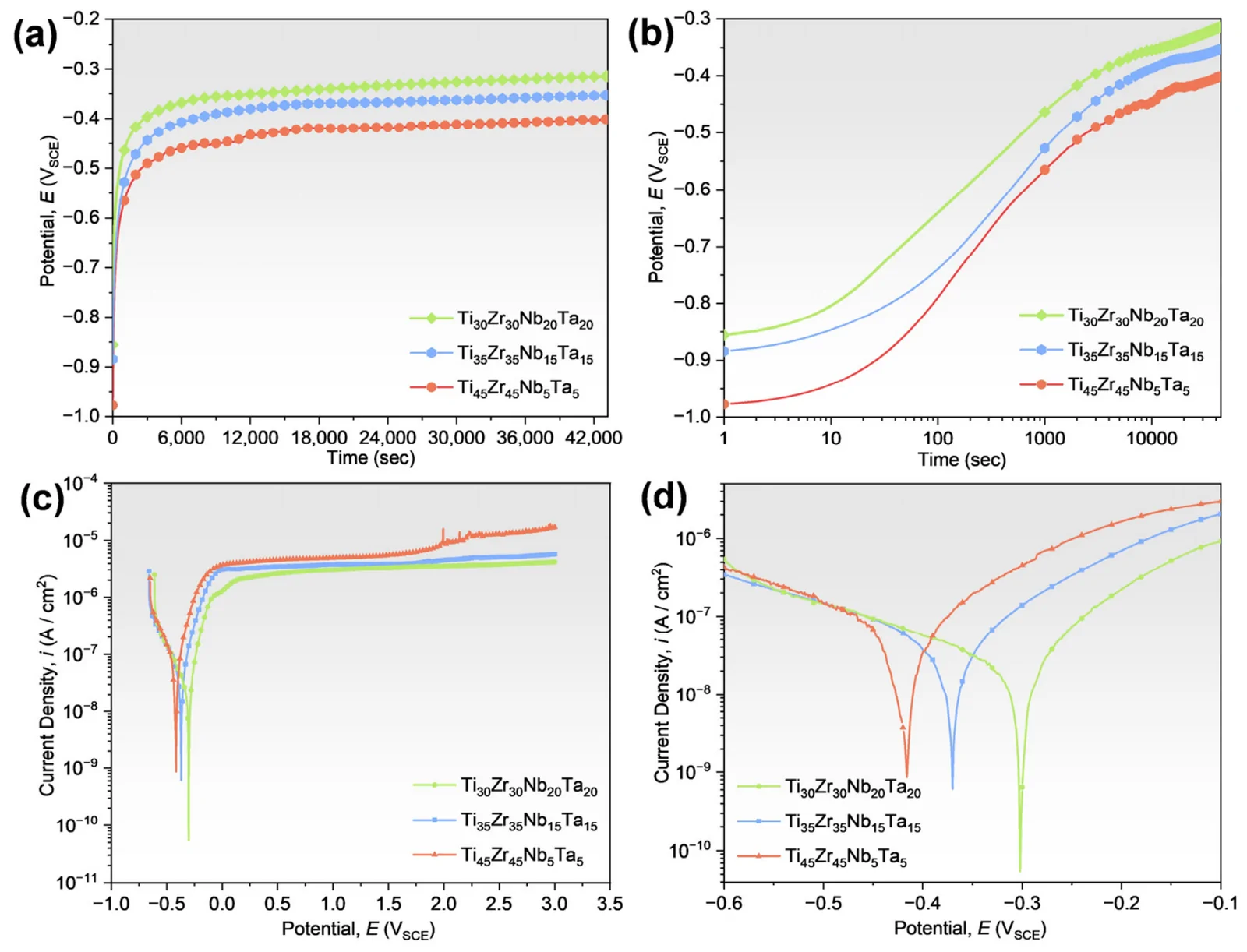
OCP evolution and PDP curves of TiZrNbTa RMPEAs immersed in 0.9 wt.% NaCl solution: (**a**) OCP evolution with immersion time; (**b**) OCP versus logarithmic immersion time; (**c**) PDP curves; (**d**) magnified Tafel regions for *i*_corr_ extraction.

Figure 3a,b presents the OCP evolution of TiZrNbTa RMPEAs as a function of immersion time in 0.9 wt.% NaCl solution. As shown in Figure 3a, the OCP of all specimens shifted positively at the initial immersion stage and gradually approached a steady-state value with prolonged immersion. Further analysis based on Figure 3b reveals that the OCP evolution can be divided into three characteristic stages. During the initial several tens of seconds, passive film nucleation occurs with a relatively low positive-shift rate of OCP. Subsequently, the passive film enters a rapid-growth regime, where OCP increases nearly linearly on the logarithmic time scale. Following this rapid-growth period, the passive film undergoes slow growth, and the positive shift in OCP is substantially decelerated. With increasing fraction of Group V elements, the OCP shifts positively. Upon reaching the steady-state condition, the OCP values are approximately −0.4 V_SCE_, −0.35 V_SCE_, and −0.31 V_SCE_, respectively. This indicates an enhancement in the stability of the as-formed passive film.

The growth rate of OCP reflects the growth rate of the passive film formed on the material surface. The relationship between OCP and passive film growth rate can be expressed as follows [43]:

(1) $$E=\mathrm{Const}.+\frac{2.303\delta }{P}\cdot \log t$$

where *E* corresponds to the OCP value, Const. is a constant term related to the material and corrosive ions in solution, *δ* represents the film-thickening rate, *P* is a constant equal to 39 nm·V^−1^ [44], and *t* is the immersion time. As illustrated in the OCP-log*t* plot in Figure 3b, two linear kinetic regimes were observed for all tested alloys, and this linear behavior complies with Equation (1). Slope analysis reveals that the kinetic rate of the rapid passive film growth regime increases with the fraction of Group IV (Ti, Zr) elements. In contrast, no significant difference in film growth rate was observed among all specimens upon entering the slow growth regime.

As shown in Figure 3c, all PDP curves pass directly from the Tafel region into a wide passive region, with no active dissolution peak being observed, indicating that the TiZrNbTa RMPEAs possess typical self-passivation behavior at the corrosion potential (*E*_corr_) [45]. The passive current density decreases with increasing Nb and Ta contents, with the values for the Ti_45_Zr_45_Nb_5_Ta_5_, Ti_35_Zr_35_Nb_15_Ta_15_, and Ti_30_Zr_30_Nb_20_Ta_20_ alloys being approximately 4.90, 3.86, and 3.05 μA·cm^−2^, respectively. For the Ti_45_Zr_45_Nb_5_Ta_5_ and Ti_35_Zr_35_Nb_15_Ta_15_ alloys, distinct current peaks appeared near 1.5 V_SCE_ on the polarization curves with increasing potential, which mainly originates from oxygen evolution triggered by electron transfer across the surface oxide film [46,47]. In contrast, no obvious oxygen evolution current peak was observed for the Ti_30_Zr_30_Nb_20_Ta_20_ alloy. This indicates that the passive film formed on Ti_30_Zr_30_Nb_20_Ta_20_ possesses superior dielectric properties and can effectively block electron transfer. Previous studies on pure Ti have confirmed that the current peak at 1.5 V_SCE_ in PDP curves arises from reduced activation energy and the enhanced diffusivity of $V_{o}^{\cdot \cdot }$ inside the passive film [48]. $V_{o}^{\cdot \cdot }$ defects within the passive film act as electron carriers, increasing the carrier concentration and migration rate and thus accelerating electron transport. It is worth noting that no abrupt and continuous current increase indicative of passive film breakdown was observed for any of the alloys, even when the polarization potential rose up to 3.0 V_SCE_. This demonstrates the excellent stability of their passive films. At *E*_corr_, the passive film for all specimens reaches steady-state balance with comparable growth and dissolution rates. Cations leached from the matrix predominantly contribute to surface oxide formation. At the steady state, the oxide film dissolution rate matches the release rate of metal cations for film growth. Hence, the measured corrosion current density (*i*_corr_) can represent the dissolution rate of surface oxide films. Figure 3d displays the Tafel regions from the PDP curves of different alloys, and Tafel extrapolation is applied to obtain the *i*_corr_. The measured *i*_corr_ obtained for the Ti_45_Zr_45_Nb_5_Ta_5_, Ti_35_Zr_35_Nb_15_Ta_15_ and Ti_30_Zr_30_Nb_20_Ta_20_ alloys are 76, 45 and 20 nA·cm^−2^, respectively.

### 3.3. Potentiostatic Polarization

**Figure 4:**
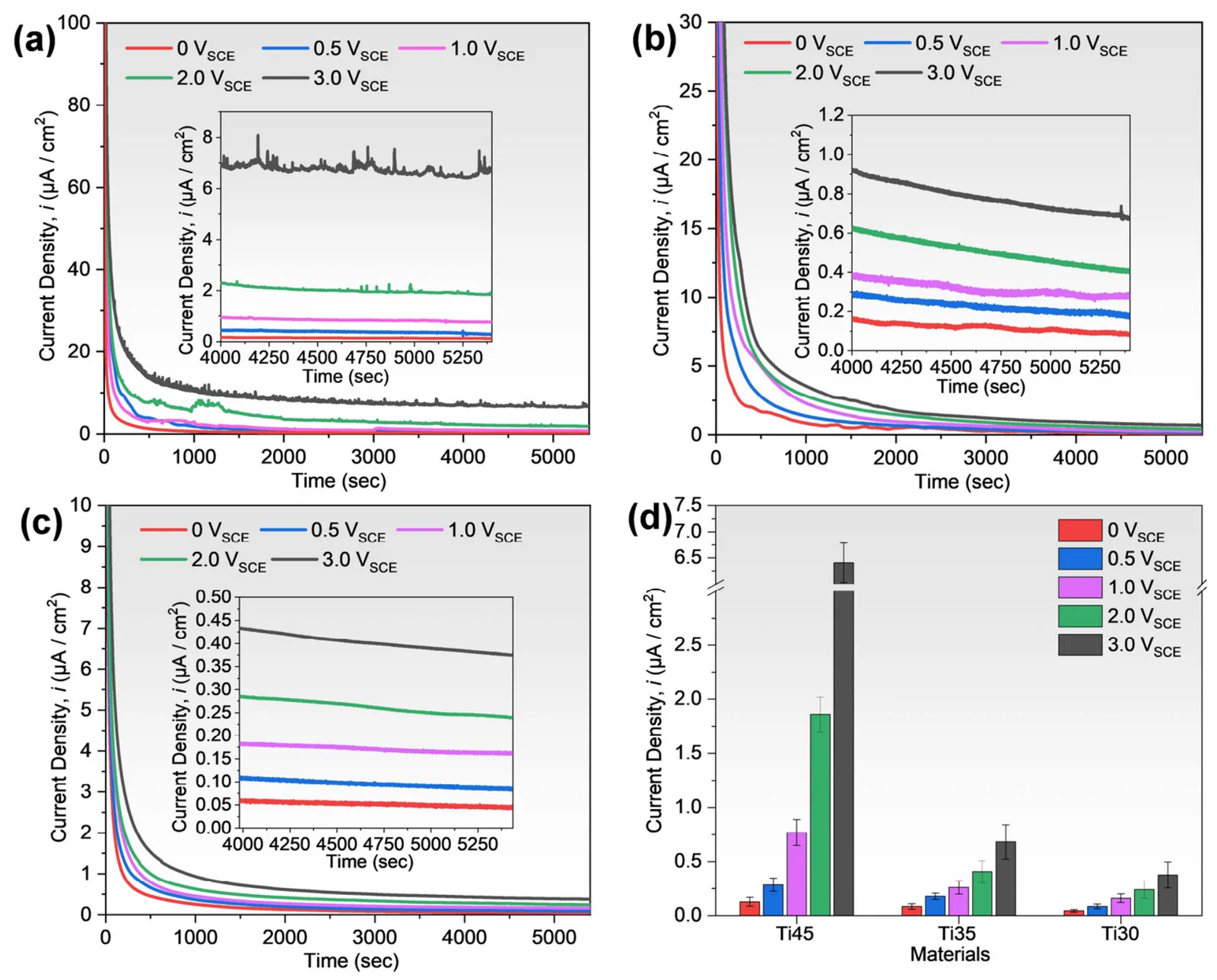
PSP curves of TiZrNbTa RMPEAs under various applied potentials: (**a**) PSP curves of the Ti_45_Zr_45_Nb_5_Ta_5_ alloy; (**b**) PSP curves of the Ti_35_Zr_35_Nb_15_Ta_15_ alloy; (**c**) PSP curves of the Ti_30_Zr_30_Nb_20_Ta_20_ alloy; (**d**) summary of *i*_ss_ for all alloys at different potentials.

To investigate the relationship between the passive films of alloys with different compositions and applied potential, PSP tests were carried out at multiple potentials selected according to the passivation region derived from PDP curves. Figure 4 shows the PSP curves of the tested alloys under different potentials. Generally, the current response during PSP indicates the processes of oxide film formation, oxide film dissolution and oxygen evolution [49]. At the initial polarization stage, a substantial rapid drop in current density is observed for all alloys, demonstrating that the current originating from the nucleation and growth of passive films is dominant at this stage. Subsequently, the current density decays to relatively steady values over time. As pointed out by Macdonald [50], this steady-state phenomenon arises from the simultaneous occurrence of oxide film formation and dissolution, achieving an equilibrium state theoretically. In practice, the current density still decreases slightly over time, and theoretical steady-state passivation is difficult to achieve [51,52]. Therefore, for the purposes of investigation, the system is typically considered to be under quasi-steady-state conditions [53]. It can be observed that the *i*_ss_ increases as the applied potential increases from 0 V_SCE_ to 3.0 V_SCE_. Distinct current spikes and fluctuations emerge on the potentiostatic curves of Ti_45_Zr_45_Nb_5_Ta_5_ at 2.0 V_SCE_ and 3.0 V_SCE_, which correspond to repeated nucleation, growth and repassivation of metastable pits [54]. This observation is consistent with the PDP results, indicating poorer passive film stability of this alloy compared with the other compositions. Based on the test results in Figure 4a–c, the *i*_ss_ of alloys with different compositions under various applied potentials are summarized and plotted in Figure 4d. It can be seen that the *i*_ss_ decreases significantly with increasing Nb and Ta contents, which implies a concomitant improvement in the stability of the passive film.

### 3.4. Electrochemical Impedance Spectroscopy

**Figure 5:**
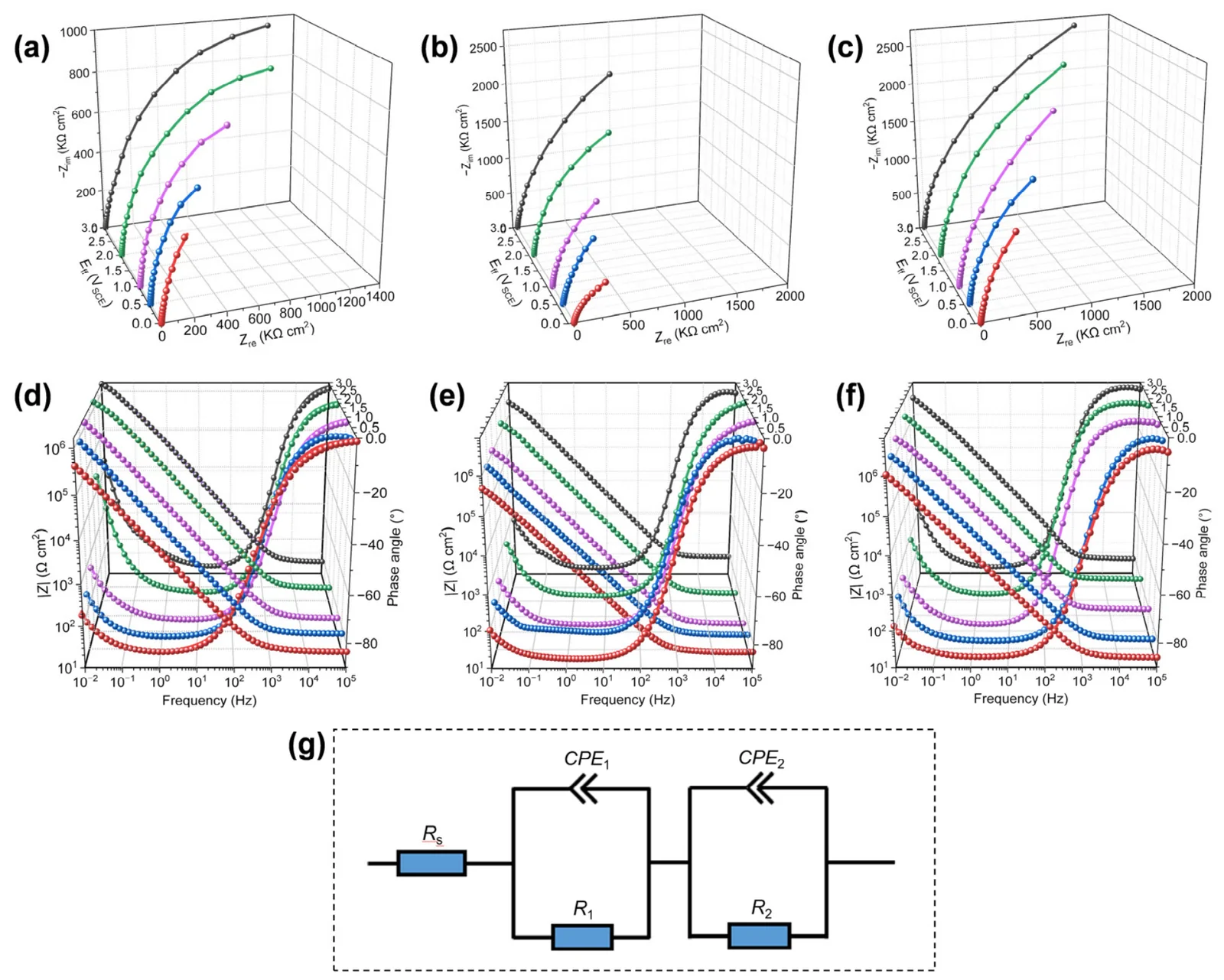
EIS results of the TiZrNbTa RMPEAs measured at different applied potentials and the corresponding EEC: (**a**–**c**) Nyquist plots of the Ti_45_Zr_45_Nb_5_Ta_5_, Ti_35_Zr_35_Nb_15_Ta_15_, and Ti_30_Zr_30_Nb_20_Ta_20_ alloys, respectively. The red, blue, magenta, green, and black curves correspond to applied potentials of 0, 0.5, 1.0, 2.0, and 3.0 V_SCE_, respectively; *Z*_re_ denotes the real part of impedance; (**d**–**f**) Bode plots of the Ti_45_Zr_45_Nb_5_Ta_5_, Ti_35_Zr_35_Nb_15_Ta_15_, and Ti_30_Zr_30_Nb_20_Ta_20_ alloys, respectively. The red, blue, magenta, green, and black curves correspond to applied potentials of 0, 0.5, 1.0, 2.0, and 3.0 V_SCE_, respectively; (**g**) EEC for EIS fitting.

After the PSP tests, EIS tests were performed at several selected potentials within the passive region to investigate the resistive properties of the as-formed passive films. Figure 5a–c presents the representative Nyquist plots for the three alloys, which exhibit truncated semicircles. The radii of these capacitive arcs are related to the polarization resistance of passive films and vary among the investigated alloys. With increasing applied potential, the diameter of the semicircular arcs exhibited a slight increase. However, the increase in semicircle diameter was more pronounced with rising Nb and Ta contents, indicating enhanced resistance of the passive films. Flattened capacitive arcs were observed in the high-frequency (HF) region, reflecting the frequency dispersion effect arising from surface heterogeneity. Due to the exceptionally high polarization resistance of the system, the low-frequency impedance tails did not converge to the real axis in the present measurements. In the Bode plots (Figure 5d–f), the |Z| value remains constant and the phase angle approaches 0° in the HF region (5–100 kHz), reflecting the response of the electrolyte resistance. The impedance modulus values derived from the linear slopes are all close to −1, while the phase angles in the mid-frequency (MF) and low-frequency (LF) regions approach 80°, indicating highly capacitive behavior, which implies the formation of a highly stable passive film on the specimen surface. The two time constants corresponding to the phase angle maxima in the MF and LF regions overlap with each other, manifesting as broadening [55] and asymmetry [56] of the phase angle curves. The EIS results were analyzed using the equivalent electrical circuit (EEC) commonly employed for TiZrNbTa RMPEAs in previous studies. The passive films on the alloys consisted of a compact inner layer and a porous outer layer, as illustrated in Figure 5g. The fitting results are presented in Tables S1–S3, with reasonable values obtained for both the chi-squared (χ^2^) and the fitting parameters. *R*_s_ represents the solution resistance, and the constant-phase element (*CPE*) is adopted to describe the non-ideal capacitive effect originating from the relaxation-time distribution induced by electrode surface heterogeneity. The HF response is dominated by the *CPE*_1_-*R*_1_ time constant, which corresponds to the resistive-capacitive behaviour of the outer porous film and is associated with the dissolution–precipitation process of the passive film. The *CPE*_2_-*R*_2_ at LF reflects the dielectric properties of the inner barrier layer [57]. Its impedance is expressed as $Z_{\mathrm{CPE}}={\left[Q{\left(\mathrm{j\omega}\right)}^{n}\right]}^{-1}$, where *j* is the imaginary unit, *ω* is the angular frequency, *Q* is the *CPE* magnitude, and *n* is the *CPE* dispersion index (0 ≤ *n* ≤ 1).

According to the parameters listed in Tables S1–S3, *R*_2_ is much larger than *R*_1_, demonstrating that the protective performance of the passive film is mainly governed by the inner barrier layer. According to the power-law distribution model proposed by Hirschorn et al. [58], this model assumes that the resistivity follows a normal distribution along the film thickness and that the dielectric constant is independent of position. It has been demonstrated to be a more accurate approach for calculating the effective capacitance (*C*_eff_) of passive films and can be expressed as:

(2) $$C_{\mathrm{eff}}=\mathrm{gQ}{\left(\rho_{\delta }\varepsilon_{r}\varepsilon_{0}\right)}^{1-n}$$

where *g* = 1 + 2.88(1 − *n*)^2.375^ is a function of *n*, *ρ*_*δ*_ is the lower interfacial resistivity bound (set to 2 × 10^7^ Ω·cm^2^ [58]), and *ε*_0_ is the vacuum permittivity (8.854 × 10^−14^ F·cm^−1^). The dielectric constant (*ε*_r_) values of the passive films on TiZrNbTa RMPEAs were obtained via the logarithmic mixing rule ($\ln\varepsilon_{r}=\sum V_{i}\ln\varepsilon_{i}$) [59], where *V*_i_ and *ε*_i_ are the volume fraction and dielectric constant of each oxide component and using $\varepsilon_{\mathrm{Ti}O_{2}}$ = 60, $\varepsilon_{Nb_{2}O_{5}}$ = 41, $\varepsilon_{Ta_{2}O_{5}}$ = 21, and $\varepsilon_{\mathrm{Zr}O_{2}}$ = 25, with *V*_i_ approximated as the elemental fraction in the film. Based on XPS analysis, the calculated *ε*_r_ values for the three alloys were 38, 36, and 34, respectively.

**Figure 6:**
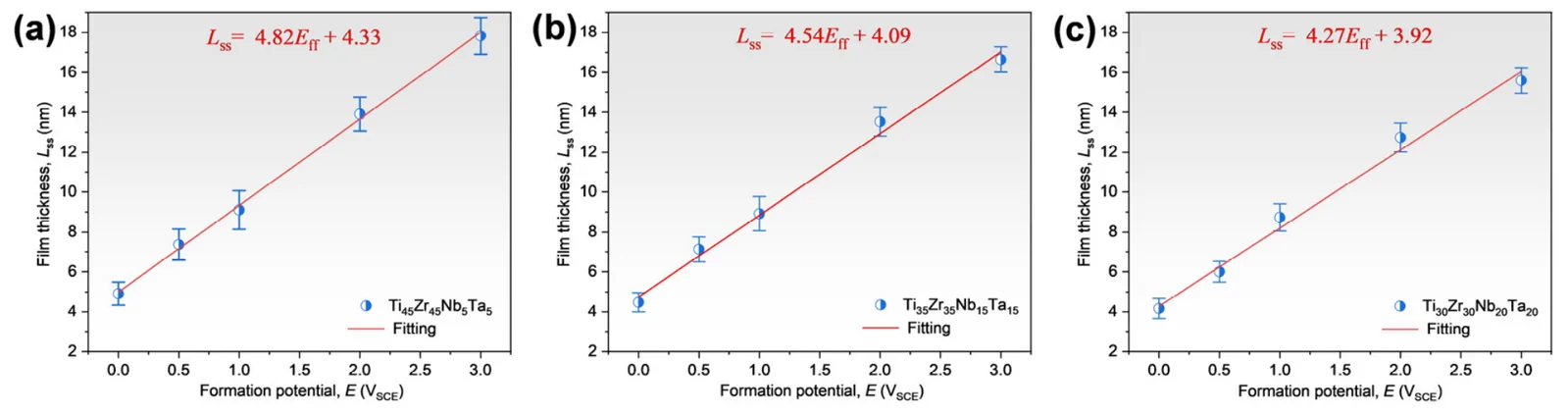
Variation of passive film thickness with formation potential and linear fitting results for the TiZrNbTa RMPEAs: (**a**) Ti_45_Zr_45_Nb_5_Ta_5_ alloy; (**b**) Ti_35_Zr_35_Nb_15_Ta_15_ alloy; (**c**) Ti_30_Zr_30_Nb_20_Ta_20_ alloy.

As revealed by the fitted parameters in Tables S1–S3, the inner compact layer exhibits smaller equivalent capacitance values (*Q*_2_) than the outer loose layer, indicating that the inner layer constitutes the major thickness contribution. The *C*_eff_ of each passive film can be calculated from Equation (2), and the thickness of the passive films formed at different potentials can then be determined by the following equation:

*L*_ss_ = (ε_r_ε_0_*A*)/*C*_eff_ (3)

where *ε*_r_ and *ε*_0_ have the same meanings as in Equation (2); A is the effective passivation area of the electrode. The calculated thicknesses of the inner barrier layers formed on each alloy at various potentials after PSP are also summarized in Tables S1–S3 and plotted in Figure 6. The field strength (*E*_0_) in the passive film and *L*_ss_ satisfy a linear functional relationship [60]:

(4) $$L_{\mathrm{ss}}=\frac{(1-\alpha )E_{\mathrm{ff}}}{E_{0}}+B$$

where *α* denotes the polarizability of the film/solution (f/s) interface (*α* = 0.5 was adopted in this work [61,62]), *E*_ff_ is the applied potential, and *B* is a constant. Based on Equation (4), linear fitting was performed for the *L*_ss_ values obtained at different *E*_ff_, as illustrated in Figure 6. The calculated *E*_0_ values of the passive films formed on the Ti_45_Zr_45_Nb_5_Ta_5_, Ti_35_Zr_35_Nb_15_Ta_15_, and Ti_30_Zr_30_Nb_20_Ta_20_ alloys were approximately 1.04 × 10^6^, 1.10 × 10^6^, and 1.17 × 10^6^ V·cm^−1^, respectively. In general, the *E*_0_ within passive films in aqueous solutions ranges from 1 to 4 MV·cm^−1^ [56,61], which is consistent with the results acquired in the present work. Notably, as the Nb and Ta contents in the alloys increase, the slope decreases progressively from 4.82 to 4.27 nm·V^−1^, indicating that the film thickening rate per unit formation potential increases with the Ti and Zr contents. In other words, alloys with higher Ti and Zr contents exhibit faster passive film growth kinetics.

### 3.5. Mott-Schottky Approach

**Figure 7:**
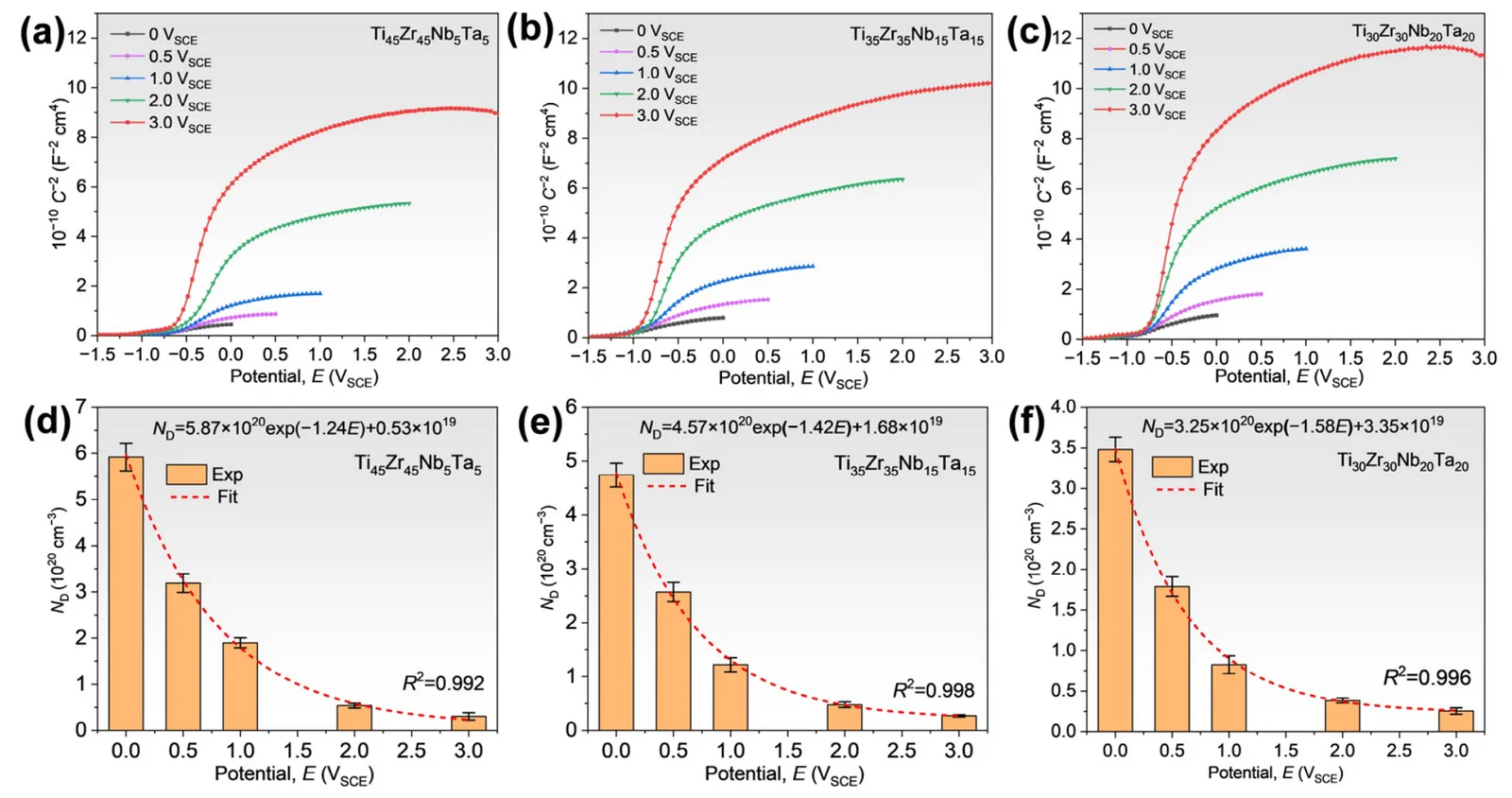
Mott–Schottky plots and calculated donor density of passive films for TiZrNbTa RMPEAs at different formation potentials: (**a**–**c**) Mott–Schottky plots of the Ti_45_Zr_45_Nb_5_Ta_5_, Ti_35_Zr_35_Nb_15_Ta_15_, and Ti_30_Zr_30_Nb_20_Ta_20_ alloys, respectively; (**d**–**f**) *N*_D_ and fitted curves of the Ti_45_Zr_45_Nb_5_Ta_5_, Ti_35_Zr_35_Nb_15_Ta_15_, and Ti_30_Zr_30_Nb_20_Ta_20_ alloys, respectively.

The semiconductor properties of passive films on alloys with different compositions under various applied potentials *E*_ff_ were investigated by Mott–Schottky measurements, as shown in Figure 7a–c. According to Mott–Schottky theory, the space-charge capacitance under the depletion-layer condition can be calculated using the following equation [6]:

(5) $$C_{\mathrm{sc}}^{-2}=\frac{2}{e\varepsilon_{r}\varepsilon_{0}N_{D}}\left(E_{\mathrm{ff}}-E_{\mathrm{fb}}-\frac{k_{b}T}{e}\right)$$

where *C*_SC_ is the electrochemical capacitance at the f/s interface; *e* is the elementary charge (1.6 × 10^−19^ C); *N* is the density of the charge carriers (*N*_A_ for acceptors and *N*_D_ for donors); *E*_fb_ denotes the flat-band potential; *k*_b_ is the Boltzmann constant (1.38 × 10^−23^ J·K^−1^); *T* is the absolute temperature. Linear fitting of the Mott–Schottky plots yields *N*_D_ from the slope:

(6) $$N_{D}=\frac{2}{e\varepsilon_{r}\varepsilon_{0}}{\left[\frac{d\left(C_{\mathrm{SC}}^{-2}\right)}{dE_{\mathrm{ff}}}\right]}^{-1}$$

All alloys exhibited a linear region with a positive slope in their Mott–Schottky plots, indicating that the passive films possess n-type semiconductor character, with oxygen vacancies ($V_{o}^{\cdot \cdot }$) as the dominant internal defects [63]. As the potential increases, the slope of the linear region of the test curve rises. According to the calculation results from Equation (6), this indicates a decreased defect concentration within the passive film, as presented in Figure 7d–f. Elevating the film-formation potential not only intensifies the consumption of $V_{o}^{\cdot \cdot }$ at the f/s interface but also promotes film thickening and ordering, thereby providing space for defect rearrangement and reducing their concentration [64,65]. The increased Nb and Ta contents reduce the concentration of $V_{o}^{\cdot \cdot }$ in the passive film, effectively impeding ionic transport within the barrier layer, which is consistent with the increasing trend of the *R*_2_ values derived from EIS fitting.

### 3.6. Analysis of Defect Diffusion in the Passive Film

The flux of point defects is critical to the growth and breakdown of passive films. Based on the PDM, the diffusion coefficient (*D*_o_) quantitatively characterizes, at the microscopic level, the transport of point defects within the film. As reported in the literature, the passive film exhibits an electric field strength on the order of 10^6^ V·cm^−1^. At this elevated field strength, the *D*_o_ is obtainable from Equation (7) [53,66]:

(7) $$D_{o}=\frac{\mathrm{z\alpha}i_{\mathrm{ss}}}{e\omega_{2}\exp\left(\mathrm{z\alpha}E_{0}F/\mathrm{RT}\right)}$$

where *z* is the charge number of the mobile point defects (*z* = 2 is used in this work), α is the half-jump distance (α = 0.4 nm [67]), *F* represents the Faraday constant (96,485 C·mol^−1^), *R* is the gas constant (8.314 J·K^−1^·mol^−1^), and *E*_0_ represents the mean electric field strength within the passive film. *ω*_1_, *ω*_2_ and *b* are the fitted constants obtained from exponential regression of the *N*_D_–*E*_ff_ curve using a first-order exponential decay function [66]:

*N*_D_ = *ω*_1_*exp*(−*bE*_ff_) + *ω*_2_ (8)

**Figure 8:**
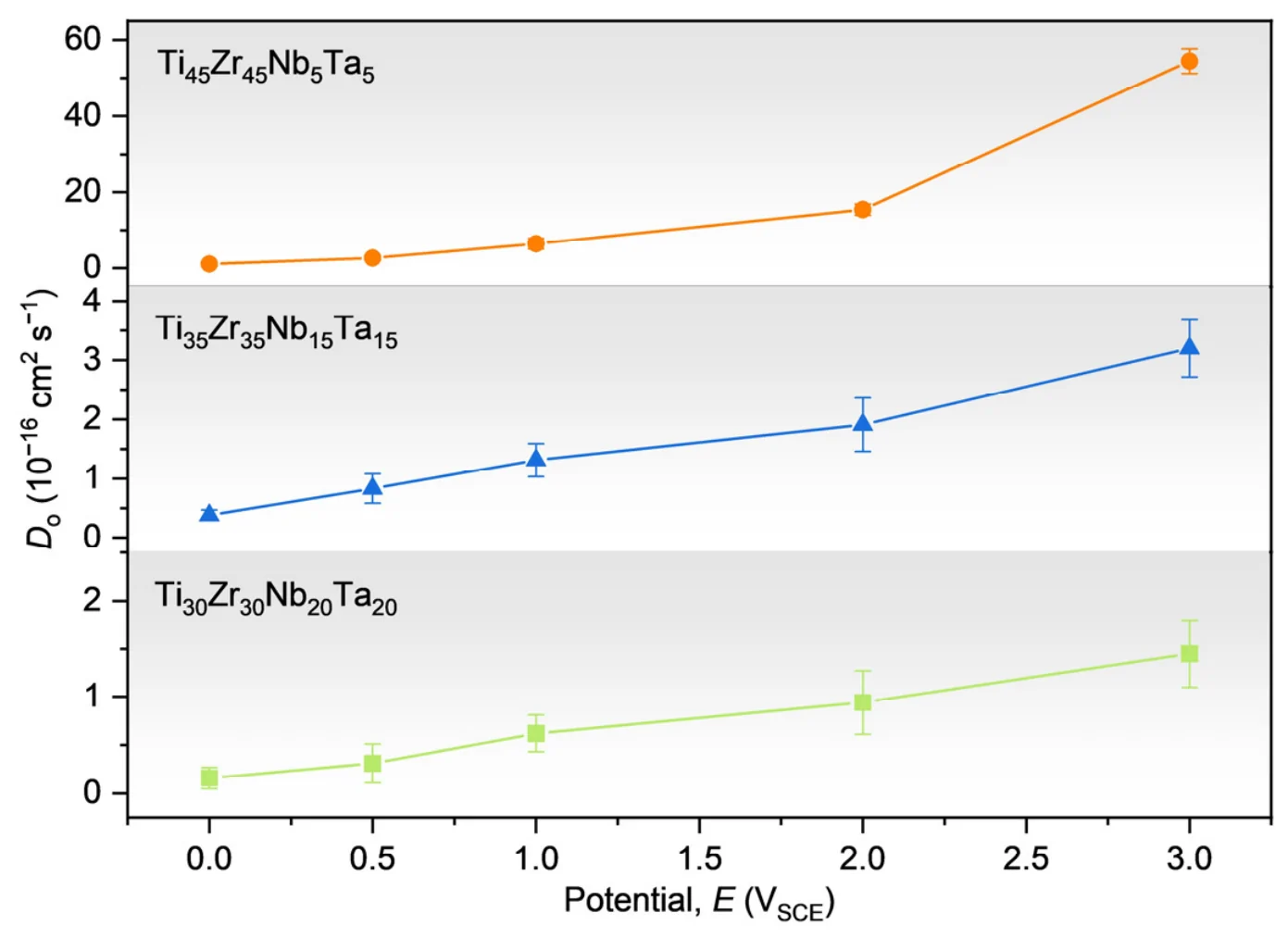
The calculated *D*_o_ of passive films formed on the Ti_45_Zr_45_Nb_5_Ta_5_, Ti_35_Zr_35_Nb_15_Ta_15_, and Ti_30_Zr_30_Nb_20_Ta_20_ alloys under various film formation potentials.

Figure 7d–f shows the *N*_D_–*E*_ff_ plots and their corresponding exponential fitting curves for the passive films formed on the tested specimens. The *ω*_2_ values for the passive films on the Ti_45_Zr_45_Nb_5_Ta_5_, Ti_35_Zr_35_Nb_15_Ta_15_, and Ti_30_Zr_30_Nb_20_Ta_20_ alloys are 0.53 × 10^19^, 1.68 × 10^19^, and 3.35 × 10^19^ cm^−3^, respectively. The relevant parameters were substituted into Equation (7) to calculate the *D*_o_ of point defects within the passive film, with the resulting data presented in Figure 8 and Tables S4–S6. Among all of the tested alloys, the *D*_o_ of point defects increases with increasing potential. Compared with the other two alloys with higher Nb and Ta contents, the Ti_45_Zr_45_Nb_5_Ta_5_ alloy exhibited a more pronounced increase in *D*_o_ above 1.0 V_SCE_. This is mainly attributed to the fact that when the applied potential exceeds 1.23 V [68], water decomposition reactions are more likely to occur on the surface of the passive film of the Ti_45_Zr_45_Nb_5_Ta_5_ alloy, thereby accelerating the generation and migration of $V_{o}^{\cdot \cdot }$ within the passive film. Additional Nb and Ta alloying markedly decreases the *D*_o_ of the passive film, indicative of improved film stability. A higher *D*_o_ corresponds to faster passive film growth kinetics, which agrees with the thickness analysis of quasi-steady-state passive films on different alloys (Figure 6). According to the PDM, the *i*_ss_ of n-type semiconductor passive films is related to the *N*_D_ and *D*_o_ within the film, as expressed below [53,66]:

*i_ss_* = 4*FKD*_o_*N*_D_ (9)

where *K* is equal to $\frac{E_{0}F}{\mathrm{RT}}$. Therefore, the *i*_ss_ of the TiZrNbTa RMPEAs, which increases with potential and with Ti and Zr content, is simultaneously attributed to the increased *N*_D_ and *D*_o_ in the passive film.

### 3.7. Potential of Zero Charge (PZC) Measurements

**Figure 9:**
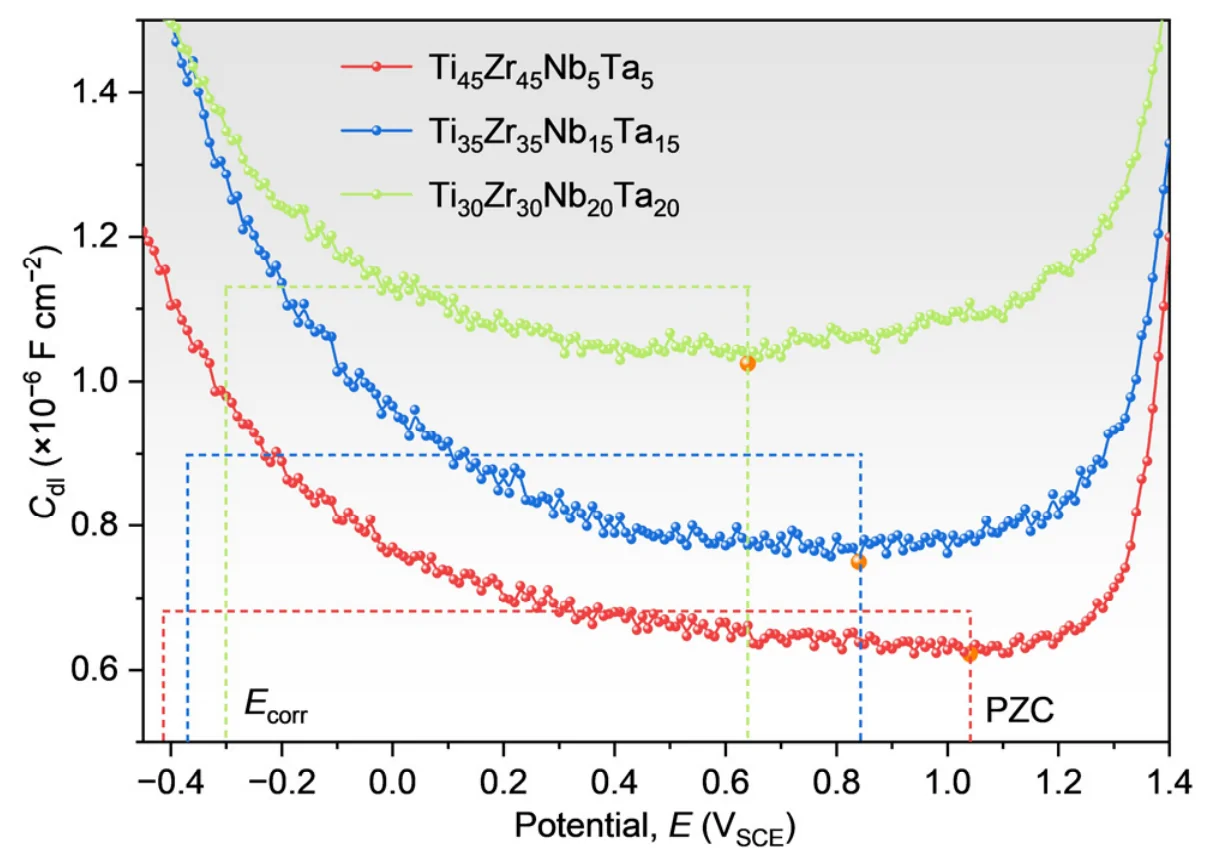
The Cdl versus potential curves and measured PZC of passive films formed on the Ti_45_Zr_45_Nb_5_Ta_5_, Ti_35_Zr_35_Nb_15_Ta_15_, and Ti_30_Zr_30_Nb_20_Ta_20_ alloys under the OCP condition. Orange dots denote the PZC points, and the width of the dotted-line box corresponds to Δ*E* = | *E*_corr_ − PZC |.

The adsorption behavior of Cl^−^ can be assessed by measuring the PZC. According to Gouy–Chapman theory, the *C*_dl_–*E* curve (double-layer capacitance versus potential) exhibits a pronounced minimum at the PZC [69,70]. Potentials above the PZC promote excess cation adsorption on the surface, whereas those below the PZC favor excess anion adsorption. Figure 9 presents the *C*_dl_–*E* curves of the three alloys, from which the PZC values of the Ti_45_Zr_45_Nb_5_Ta_5_, Ti_35_Zr_35_Nb_15_Ta_15_, and Ti_30_Zr_30_Nb_20_Ta_20_ alloys were determined to be 1.05, 0.83, and 0.64 V_SCE_, respectively. Notably, the *E*_corr_ of all three alloys lies negative of their respective PZC, indicating spontaneous Cl^−^ adsorption on every sample surface. The potential difference between *E*_corr_ and the PZC (Δ*E* = | *E*_corr_ − PZC |) was 1.46, 1.20, and 0.94 V for the Ti_45_Zr_45_Nb_5_Ta_5_, Ti_35_Zr_35_Nb_15_Ta_15_, and Ti_30_Zr_30_Nb_20_Ta_20_ alloys, respectively. A larger | *E*_corr_ − PZC | (i.e., a potential more negative relative to the point of zero charge) corresponds to a stronger driving force for Cl^−^ adsorption [71,72]. Accordingly, increasing the Nb and Ta contents can markedly suppress Cl^−^ adsorption on the surface of the alloy passive film, and this trend is consistent with the metastable pitting behavior shown in Figure 4.

### 3.8. XPS Analysis of Passive Film

**Figure 10:**
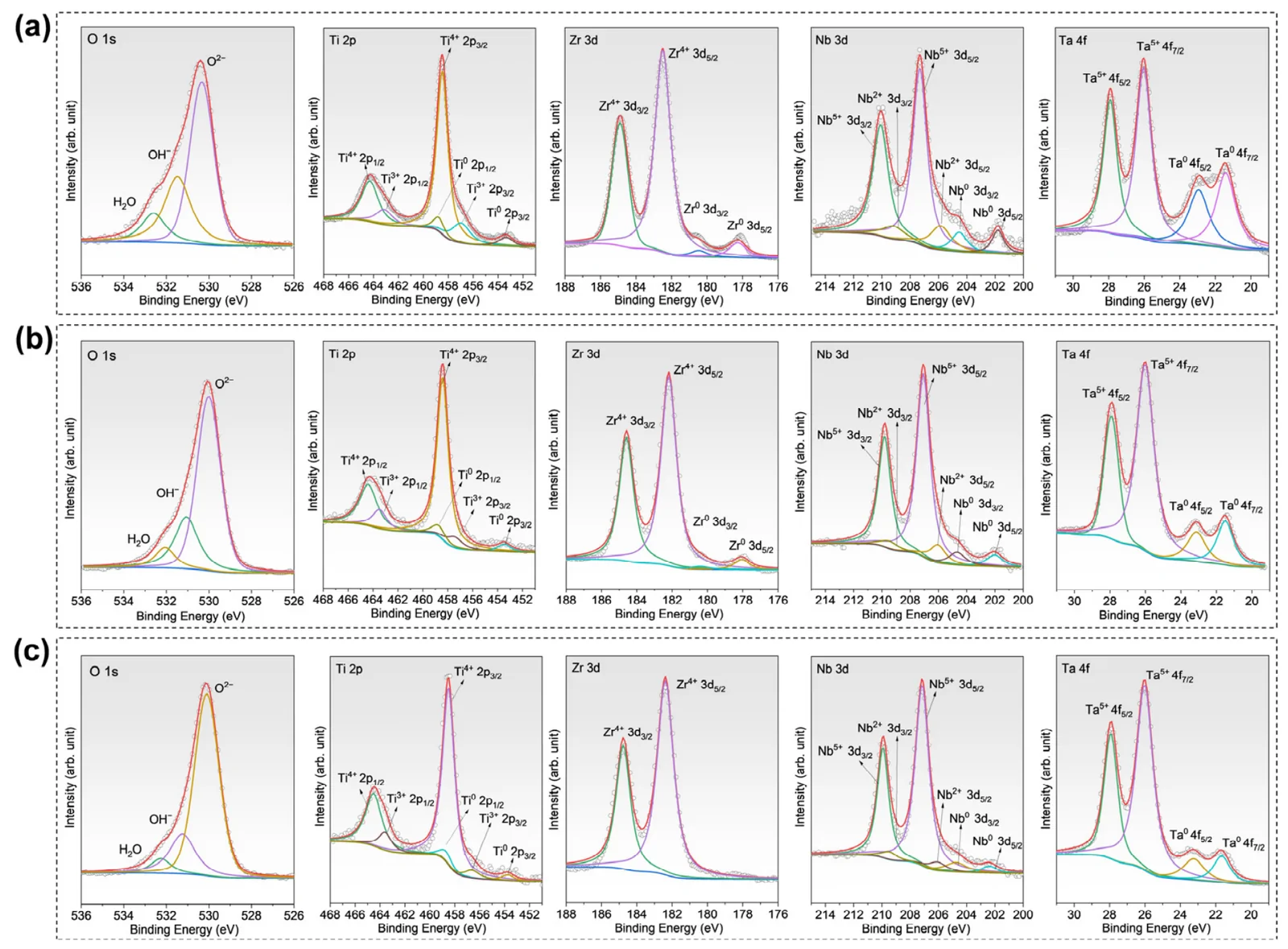
High resolution XPS spectra of O 1s, Ti 2p, Zr 3d, Nb 3d and Ta 4f for passive films formed on TiZrNbTa RMPEAs under the OCP condition: (**a**) Ti_45_Zr_45_Nb_5_Ta_5_ alloy; (**b**) Ti_35_Zr_35_Nb_15_Ta_15_ alloy; (**c**) Ti_30_Zr_30_Nb_20_Ta_20_ alloy.

**Figure 11:**
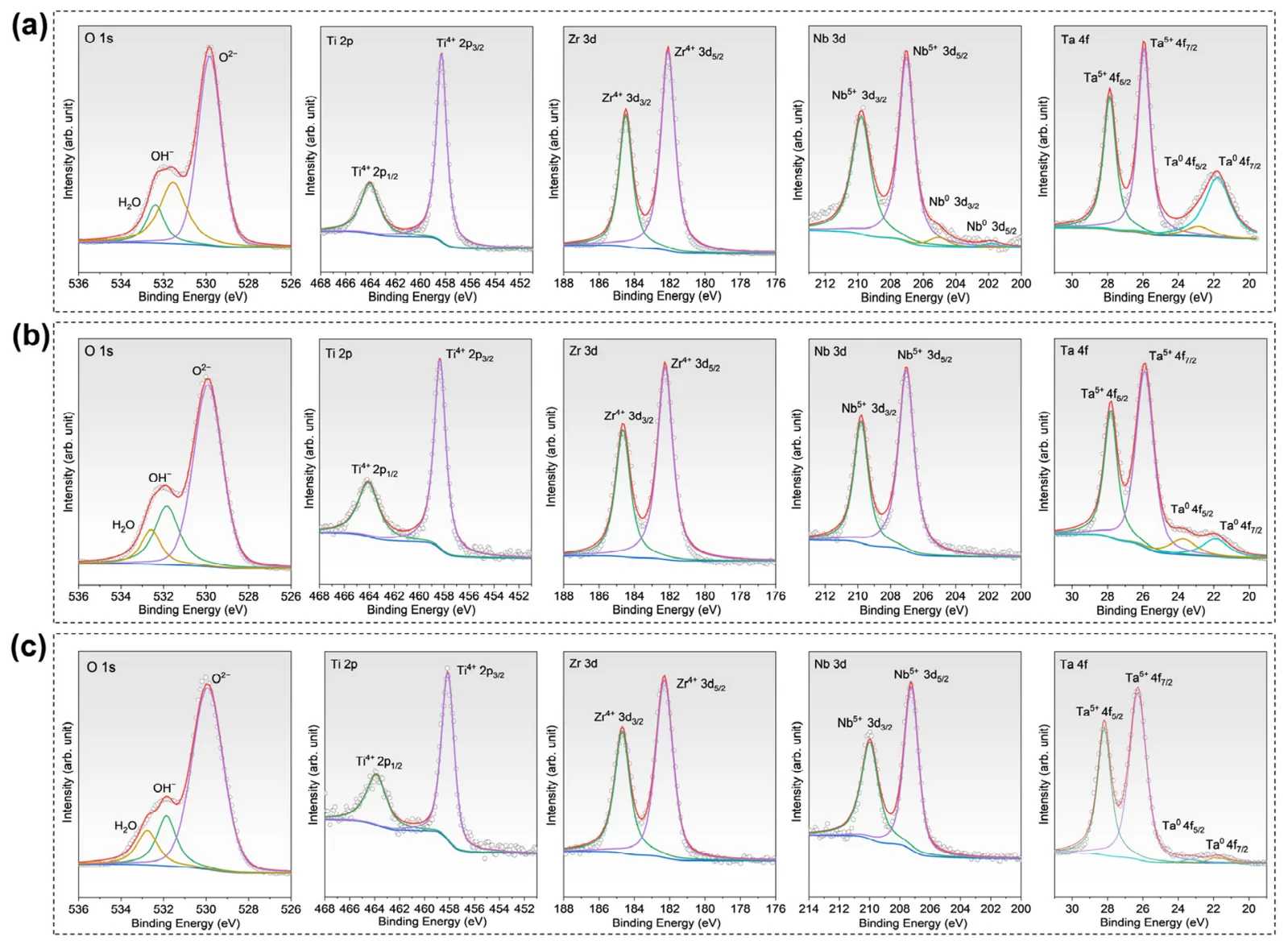
High resolution XPS spectra of O 1s, Ti 2p, Zr 3d, Nb 3d and Ta 4f for passive films formed on TiZrNbTa RMPEAs at an applied potential of 3.0 V_SCE_: (**a**) Ti_45_Zr_45_Nb_5_Ta_5_ alloy; (**b**) Ti_35_Zr_35_Nb_15_Ta_15_ alloy; (**c**) Ti_30_Zr_30_Nb_20_Ta_20_ alloy.

**Figure 12:**
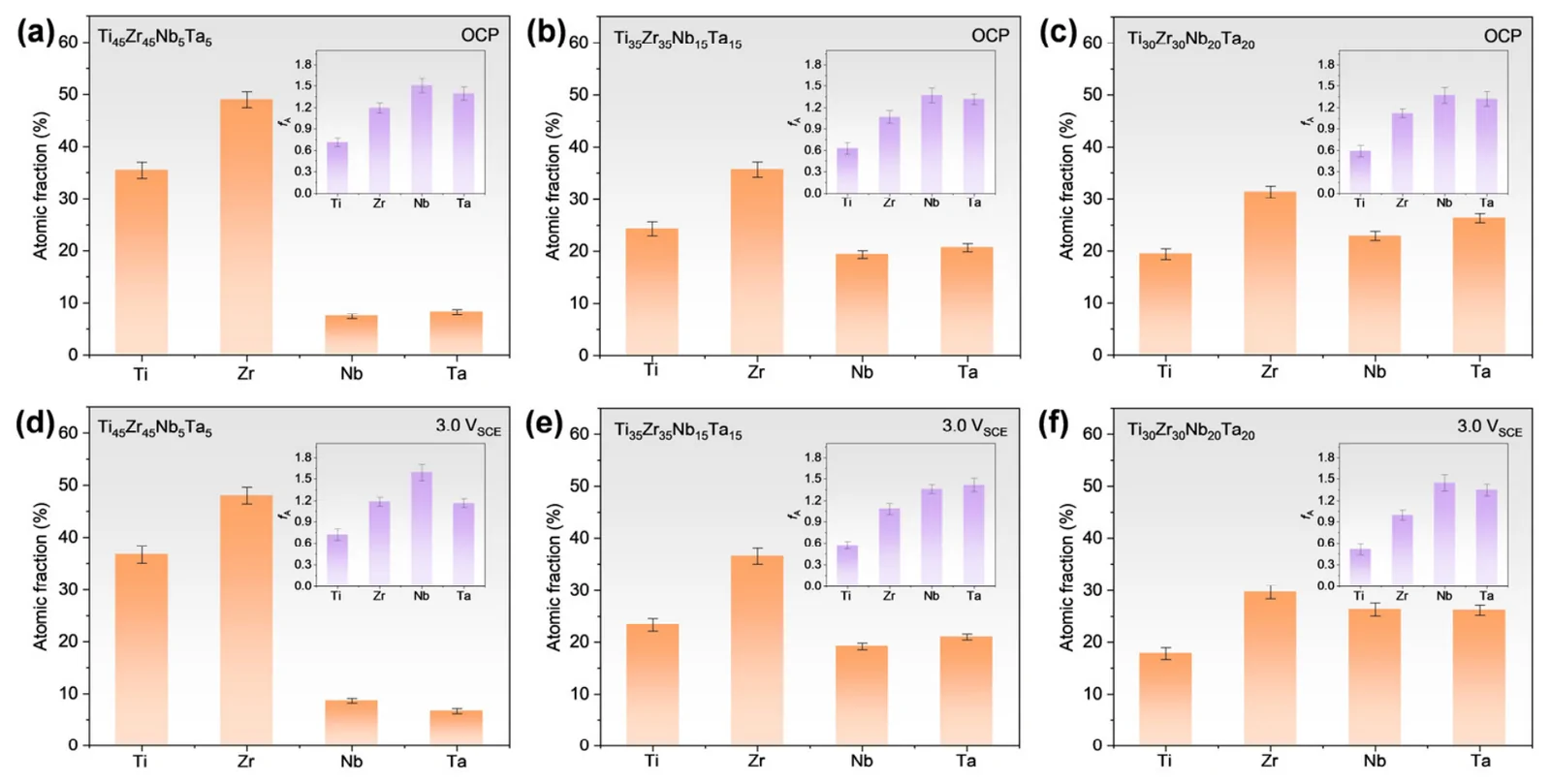
Atomic fraction of elements and surface enrichment factor *f*_A_ in passive films formed on TiZrNbTa RMPEAs under OCP and 3.0 V_SCE_: (**a**–**c**) OCP condition for the Ti_45_Zr_45_Nb_5_Ta_5_, Ti_35_Zr_35_Nb_15_Ta_15_, and Ti_30_Zr_30_Nb_20_Ta_20_ alloys, respectively; (**d**–**f**) 3.0 V_SCE_ condition for the Ti_45_Zr_45_Nb_5_Ta_5_, Ti_35_Zr_35_Nb_15_Ta_15_, and Ti_30_Zr_30_Nb_20_Ta_20_ alloys, respectively.

Figure 10 and Figure 11 display the high-resolution XPS spectra of oxygen and all constituent elements in the passive films formed on the tested alloys under OCP and 3.0 V_SCE_, respectively. The O 1s spectrum is fitted with three components assigned to lattice oxygen (O^2−^, i.e., oxygen anions bound in metal oxides), hydroxide/hydroxyl species (OH^−^), and adsorbed surface water, indicating substantial hydration in the outermost layer of the passive film. Combined with the EIS results, the hydroxyl groups originate from the porous oxide outer layer formed by the dissolution of the passive film itself [73]. The O^2−^ species dominates, and the proportions of OH^−^ and surface adsorbed water grow with increasing Ti and Zr contents. The OH^−^ peak intensity is also more pronounced under applied potential (Figure 11a–c). According to the Marcus passivity theory and elemental oxidation thermodynamic potentials [74], Ti and Zr act as passivity promoters with high adsorption heats of O^2−^ or OH^−^ during oxide nucleation and growth. In the surface region probed by XPS, the constituent elements of all samples exist predominantly as stable high-valence oxides (TiO_2_, ZrO_2_, Nb_2_O_5_, and Ta_2_O_5_). Metallic, intermediate-valence, and high-valence components of all constituent elements in the TiZrNbTa alloys were fitted with full spin–orbit doublets, and the binding energy positions of each component were assigned with reference to the published literature [75,76,77]. The comparatively lower fractions of metallic and intermediate-valence species for Ti and Zr indicate that these elements are more readily oxidized to high-valence states than Nb and Ta, which is consistent with the more negative Gibbs free energies of the oxidation of Ti and Zr [29,30]. Under OCP conditions, the passive film of the Ti_45_Zr_45_Nb_5_Ta_5_ alloy exhibited the highest overall fractions of metallic and intermediate-valence species among the three alloys. The fitting results show that increasing the Nb and Ta contents promotes the formation of stable high-valence oxides. The applied potential further promotes the oxidation of metallic and intermediate-valence species to high-valence states (Figure 11). At 3.0 V_SCE_, no detectable metallic or lower-valence Ti and Zr components remained in the passive films of any of the alloys. For Ti_35_Zr_35_Nb_15_Ta_15_ and Ti_30_Zr_30_Nb_20_Ta_20_, no lower-valence Nb components were detected either. The cation fractions and surface enrichment factors were recalculated from the fitting results and updated in Figure 12.

Based on the deconvoluted peak intensities of each element, the atomic concentrations of all components in the surface layer of the passive films for all alloys under different conditions were calculated, and the results are shown in Figure 12. It can be seen that compared with the atomic concentrations of constituents in the alloy matrix, the passive films of all samples exhibited evident Ti depletion, which is consistent with the findings reported for analogous alloy systems in the existing literature [78]. The Zr concentration was roughly comparable to that of the matrix, while Nb and Ta were markedly enriched. With increasing Nb and Ta alloying extent, the atomic concentrations of Nb and Ta in the passive film increase accordingly. From cation fractions derived from XPS analysis, the surface enrichment factor (*f*_A_) for element A can be calculated using the Castle–Asami method [11], as shown in Equation (10):

(10) fA=X A ox/X A ox+∑jXj oxX A ox/XAb+∑jXjb

where *j* denotes alloying elements other than the target element A; $X_{A}^{\mathrm{ox}}$ is the oxide fraction of element A, and $\sum_{j}X_{j}^{\mathrm{ox}}$ is the total oxide fraction of all elements except A. $X_{A}^{b}$ represents the atomic fraction of element A in the alloy matrix, while $\sum_{j}X_{j}^{b}$ is the total atomic fraction of matrix alloying elements excluding *A*. *f*_A_ > 1 signifies enrichment of element A in the analyzed region, whereas *f*_A_ < 1 denotes depletion. The numerically computed enrichment–depletion coefficients for each element in the passive films formed under different conditions are summarized in the inset subgraphs of Figure 12, offering intuitive quantitative evidence for elemental redistribution occurring during passivation. It can be seen that the *f*_A_ values of Nb and Ta are markedly greater than 1, Zr is approximately equal to 1, while Ti is distinctly lower than 1. Notably, the degree of Ti depletion in passive films is intensified with increasing Nb and Ta addition in the alloy matrix.

## 4. Discussion

At the initial stage of passive film formation, the constituent elements in the TiZrNbTa RMPEAs undergo the following reactions at the metal/solution (m/s) interface, and the equilibrium electrode potentials of the above reactions are obtained from thermodynamic data [79]:

Ti + 2H_2_O ⇌ TiO_2_ + 4H^+^ + 4e^−^           *E*^0^ = −1.490 V (11)

Zr + 2H_2_O ⇌ ZrO_2_ + 4H^+^ + 4e^−^           *E*^0^ = −1.887 V (12)

2Nb + 5H_2_O ⇌ Nb_2_O_5_ + 10H^+^ + 10e^−^    *E*^0^ = −1.015 V (13)

2Ta + 5H_2_O ⇌ Ta_2_O_5_ + 10H^+^ + 10e^−^       *E*^0^ = −1.166 V (14)

The redox equilibrium potentials of the above reactions relative to the standard hydrogen electrode show that Ti and Zr possess more negative equilibrium potentials, which makes them more susceptible to oxidation to preferentially form stable surface oxide layers and dominate the rapid oxide growth. Accordingly, alloys with higher Ti and Zr contents, such as the Ti_45_Zr_45_Nb_5_Ta_5_ alloy, exhibit a more rapid increase in OCP during passive film growth, yet their OCP remains relatively negative (Figure 3a,b). The faster film formation of the Ti/Zr-rich alloys is further corroborated by the potential dependence of the quasi-steady-state film thickness (Figure 6). The increment of film thickness per unit formation potential increases from 4.27 to 4.82 nm·V^−1^ with increasing Ti and Zr additions. The thermodynamic stability of oxides can be assessed by their standard Gibbs free energy of formation, and a more negative value of this parameter indicates higher structural stability of the oxide. For the present TiZrNbTa alloy system, the surface oxidation products Nb_2_O_5_ (−1940.47 kJ·mol^−1^) and Ta_2_O_5_ (−2088.66 kJ·mol^−1^) exhibit markedly superior thermodynamic stability compared with ZrO_2_ (−1115.32 kJ·mol^−1^) and TiO_2_ (−959.74 kJ·mol^−1^) formed in the passive film [77]. Accordingly, increasing the fractions of Ta and Nb in the alloy facilitates the enrichment of Ta_2_O_5_ and Nb_2_O_5_ within the passive film, which suppresses the dissolution loss of the passive film and reduces the measured passive current density (Figure 3c and Figure 4).

A semiconductor composed of complex oxides forms on the surface of MPEAs, which can be regarded as a highly substituted doped state with multiple cations [14,80]. Experiments also show that the quasi-steady-state barrier layer thickness increases linearly with the applied potential (Figure 6), demonstrating that the PDM can characterize the diffusion and interaction of point defects within the passive film on TiZrNbTa RMPEAs [24]. During passive film formation and under quasi-steady-state conditions, Nb and Ta atoms at the metal/film (m/f) interface are oxidized and diffuse outward, attaining higher oxidation states in the outer layer, and are incorporated into the TiO_2_ oxide, where the following Kröger–Vink defect chemistry reactions occur [81]:

(15) $$4\mathrm{Nb}(m)\;+\;5Ti_{\mathrm{Ti}}^{x}(\mathrm{ox})\to 4{Nb}_{\mathrm{Ti}}^{\cdot }(\mathrm{ox})\;+\;V_{\mathrm{Ti}}^{⁗}(\mathrm{ox})\;+\;4Ti^{4+}(\mathrm{aq})\;+\;16e\prime$$

(16) $$4\mathrm{Ta}(m)+5Ti_{\mathrm{Ti}}^{x}(\mathrm{ox})\to 4{Ta}_{\mathrm{Ti}}^{\cdot }(\mathrm{ox})+V_{\mathrm{Ti}}^{⁗}(\mathrm{ox})+4Ti^{4+}(\mathrm{aq})+16e\prime$$

The Nb^5+^ and Ta^5+^ ions occupy Ti sites substitutionally, forming substitutional defects (${Nb}_{\mathrm{Ti}}^{\cdot }$ and ${Ta}_{\mathrm{Ti}}^{\cdot }$) accompanied by the preferential dissolution of some Ti^4+^ ions into the solution and the concomitant formation of Ti-site vacancies, with electrons being transferred to the alloy matrix. When high-valence cations incorporate and occupy the local environment, negatively charged Ti cation vacancies ($V_{\mathrm{Ti}}^{⁗}$) are generated concomitantly to achieve charge self-compensation. The compensating vacancies and intrinsically existing $V_{o}^{\cdot \cdot }$ within the non-stoichiometric film are gradually eliminated via the following two recombination equilibria:

(17) $$\mathrm{Ti}\left(m\right)+V_{\mathrm{Ti}}^{⁗}\left(\mathrm{ox}\right)\to Ti_{\mathrm{Ti}}^{x}\left(\mathrm{ox}\right)+4e^{\prime }$$

(18) $$V_{\mathrm{Ti}}^{⁗}(\mathrm{ox})+2V_{O}^{\cdot \cdot }(\mathrm{ox})\to \mathrm{nil}$$

The co-doping of high-valence Nb and Ta cations can simultaneously eliminate two types of intrinsic defects, namely $V_{\mathrm{Ti}}^{⁗}$ and $V_{o}^{\cdot \cdot }$, within the film. According to the solute–vacancy interaction model (SVIM) [82], part of $V_{\mathrm{Ti}}^{⁗}$ can electrostatically associate with ${Nb}_{\mathrm{Ti}}^{\cdot }$/${Ta}_{\mathrm{Ti}}^{\cdot }$ to form low-mobility complexes, which further pin the mobile vacancies. The reaction is shown below:

(19) $${Nb}_{\mathrm{Ti}}^{\cdot }+V_{\mathrm{Ti}}^{⁗}⇌\{Nb_{\mathrm{Ti}}V_{\mathrm{Ti}}{\}}^{\prime \prime \prime }$$

(20) $${Ta}_{\mathrm{Ti}}^{\cdot }+V_{\mathrm{Ti}}^{⁗}⇌\{Ta_{\mathrm{Ti}}V_{\mathrm{Ti}}{\}}^{\prime \prime \prime }$$

Ti cations rely primarily on cation vacancies for outward migration. A decrease in the concentration of such defects therefore markedly suppresses the long-range outward diffusion of Ti toward the f/s interface. Doping with Nb and Ta cations also triggers the release of Ti cations in accordance with reactions (15) and (16). These two effects act in concert and ultimately produce the observed Ti depletion within the passive film, as illustrated in Figure 12.

In accordance with PDM [83], passive film failure arises from anion-catalyzed cation vacancy aggregation at the f/s interface through Schottky pair reactions:

(21) $$\mathrm{Null}\to V_{M}^{\chi -}+\chi /2V_{o}^{\cdot \cdot }$$

This process is promoted by Cl^−^. The increase in the number of $V_{O}^{\cdot \cdot }$ facilitates the adsorption of Cl^−^ via the reaction shown below:

(22) $$Cl_{\left(\mathrm{aq}\right)}^{-}\;+\;V_{o}^{\cdot \cdot }\;+\;e\prime \to Cl_{o}^{\cdot }$$

$V_{O}^{\cdot \cdot }$ is continuously occupied and consumed by Cl^−^, which disturbs the Schottky equilibrium at the f/s interface and produces extra defect pairs according to this defect reaction (21). Experimental results indicate that increasing Nb and Ta contents in the alloy weakens the adsorption of Cl^−^ on the passive film surface (Figure 9). In addition, the presence of high contents of stable high-valence Nb and Ta oxides within the passive film can slow down the dissolution reaction of the passive film induced by the Cl^−^:

(23) $$MO_{\chi /2}+\chi H^{+}+Cl_{\left(\mathrm{aq}\right)}^{-}\to \mathrm{MC}l^{(\chi -1)+}+V_{M}^{\chi -}+\chi /2V_{o}^{\cdot \cdot }$$

**Figure 13:**
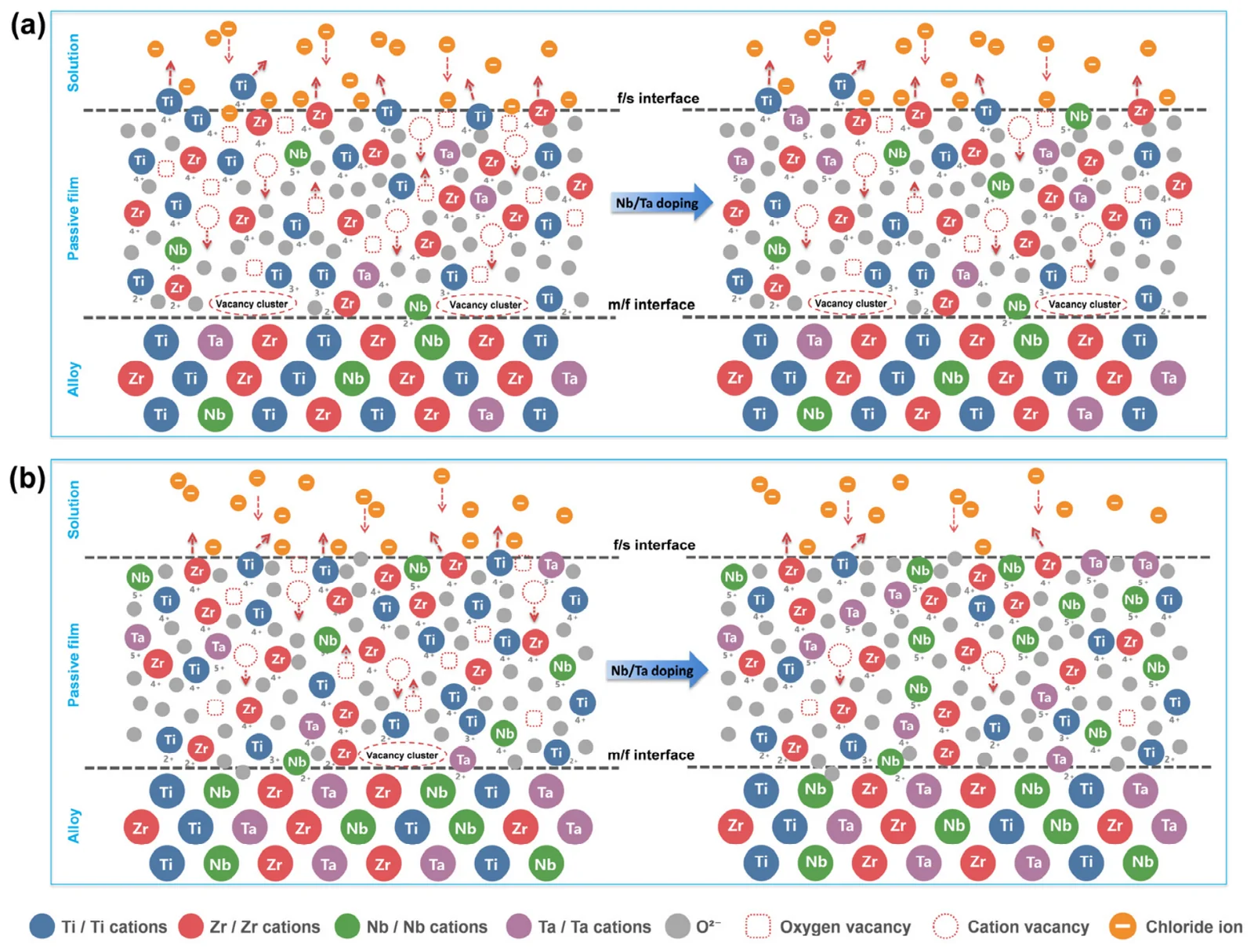
Schematic of Nb/Ta high-valence cation doping effects on point defects, diffusion, and cation dissolution in the passive film: (**a**) low content Nb/Ta doping limitedly reduces defect concentration, inhibits defect diffusion, and suppresses film dissolution induced by Cl^−^ adsorption; (**b**) high content Nb/Ta doping significantly reduces defect concentration, hinders defect transport, and suppresses film dissolution induced by Cl^−^ adsorption.

These reactions collectively suppress the generation of $V_{O}^{\cdot \cdot }$ and cation vacancies ($V_{M}^{\chi -}$) at the f/s interface, thereby helping maintain a low defect concentration within the film. The calculated results confirm that the diffusion coefficient of $V_{O}^{\cdot \cdot }$ in the passive film decreases significantly with increasing Nb and Ta additions. Therefore, this slows the transport and accumulation of vacancy defects toward the m/f interface, thereby restraining the local separation and rupture between the passive film and metal substrate. Potentiodynamic and potentiostatic polarization results both demonstrate that the metastable pitting resistance of the TiZrNbTa RMPEA improves with increasing alloying fractions of Nb and Ta. It is evident from the foregoing experiments and analyses that upon metastable pitting breakdown of the passive film, Ti and Zr, due to their strong adsorption affinity toward oxygen and hydroxide ions, govern the rapid nucleation and growth of passive film on exposed metallic surfaces. Concurrently, Nb and Ta undergo continuous oxidation to form high-valence cations that become incorporated into the passive film. This process effectively lowers the defect concentration within the film and retards defect diffusion, thereby preserving the superior stability of the passive layer. Furthermore, stable high-valence oxides of Nb and Ta accumulate at the outer region of the passive film, substantially attenuating chloride-induced film dissolution. The schematic diagram of the microscopic process mechanism by which Nb and Ta doping enhances the stability of the passive film is shown in Figure 13. Collectively, the synergism between the rapid passive film self-repair mediated by Ti and Zr and the structural compactness and dissolution resistance modulated by Nb and Ta imparts exceptional long-term pitting corrosion resistance to the TiZrNbTa RMPEAs in chloride-rich corrosive environments.

## 5. Conclusions

This work systematically investigates the effects of varying Nb and Ta contents on the passivation behavior, point defect diffusion, and metastable pitting resistance of TiZrNbTa RMPEAs in 0.9 wt.% NaCl solution, and establishes a quantitative correlation among composition, defect and property for the passive films of TiZrNbTa RMPEAs. The main findings are summarized as follows:

(1) All investigated TiZrNbTa RMPEAs exhibit a single-phase BCC structure. Increasing Nb and Ta contents refine the as-cast dendrites, decrease the lattice parameter, and enhance elemental segregation. Nb and Ta are enriched in dendrites while Zr is markedly enriched in interdendritic regions and Ti shows only a weak interdendritic enrichment tendency.
(2) All TiZrNbTa RMPEAs exhibit high stability of the passive film, with pitting potentials higher than 3.0 V_SCE_. Increasing the Ti and Zr contents in the alloy promotes the growth of the passive film on the substrate and accelerates passive film formation. In contrast, raising the Nb and Ta contents can effectively reduce the uniform corrosion rate and improve the pitting corrosion resistance of the alloy.
(3) The passive films on all TiZrNbTa RMPEAs exhibit n-type semiconductor characteristics. The donor density within the passive films is on the order of 10^20^ cm^−3^, and the oxygen vacancy diffusion coefficient falls in the range of (0.16–54) × 10^−16^ cm^2^·s^−1^. With the increase in Nb and Ta contents, the point defect concentration, oxygen vacancy diffusion coefficient, and chloride ion adsorption strength on the passive film surface decrease significantly.
(4) The outer passive films of all TiZrNbTa RMPEAs are mainly composed of high-valent oxides of the constituent elements. Increasing the Nb and Ta contents can promote the conversion of each constituent element into high-valent oxides, raise the fraction of high-valent Nb and Ta oxides in the film, enhance the thermodynamic stability of the passive film, and thereby effectively suppress chloride-induced film dissolution.
(5) Compared with the bulk alloy, the passive film exhibits Ti depletion alongside the enrichment of Nb and Ta, and the degree of Ti depletion intensifies with increasing Nb and Ta additions. Based on defect chemistry and the solute–vacancy interaction model, doping by Nb and Ta cations reduces the vacancy defect concentration within the passive film, which suppresses the outward diffusion of Ti cations.
(6) Ti and Zr can accelerate the repassivation of pitting by promoting rapid passive film growth, whereas Nb and Ta suppress pitting nucleation and propagation via reducing point defect concentration and restraining defect transport. Their synergy improves the pitting resistance of TiZrNbTa RMPEAs.

## Figures and Tables

**Table 1:** EDS compositions (at.%) of the dendritic and interdendritic regions of the TiZrNbTa RMPEAs.

Elements	Ti	Zr	Nb	Ta	Ti	Zr	Nb	Ta
	Dendritic				Interdendritic			
Ti_45_Zr_45_Nb_5_Ta_5_	44.59	44.16	5.54	5.70	45.11	45.04	5.05	4.80
Ti_35_Zr_35_Nb_15_Ta_15_	33.77	26.74	17.00	22.49	36.40	38.10	13.10	12.40
Ti_30_Zr_30_Nb_20_Ta_20_	28.53	21.54	23.89	26.04	32.34	35.61	17.25	15.25

## Data Availability

The original contributions presented in this study are included in the article/Supplementary Materials. Further inquiries can be directed to the corresponding author.

## Supplementary Materials

The following supporting information can be downloaded at: https://www.mdpi.com/article/10.3390/ma19214720/s1. Table S1: Fitting parameters of the EIS measurements for the passive films formed on the Ti_45_Zr_45_Ta_5_Nb_5_ alloy at different potentials; Table S2: Fitting parameters of the EIS measurements for the passive films formed on the Ti_35_Zr_35_Ta_15_Nb_15_ alloy at different poten-tials; Table S3: Fitting parameters of the EIS measurements for the passive films formed on the Ti_30_Zr_30_Ta_20_Nb_20_ alloy at different potentials; Table S4: Diffusion coefficients (*D*_o_) of point defects in the passive film formed on the Ti_45_Zr_45_Ta_5_Nb_5_ alloy at different potentials; Table S5: Diffusion coefficients (*D*_o_) of point defects in the passive film formed on the Ti_35_Zr_35_Ta_15_Nb_15_ alloy at different potentials; Table S6: Diffusion coefficients (*D*_o_) of point defects in the passive film formed on the Ti_30_Zr_30_Ta_20_Nb_20_ alloy at different potentials.
