# Supplementary material for: Role of Niobium and Tantalum on the Diffusivity of Point Defects in the Passive Film of TiZrNbTa Multi-Principal Element Alloys in Sodium Chloride Solution
Source: Materials (Basel). 2026 Nov 6;19(21):4720. doi: 10.3390/ma19214720 (PMC13765260; doi:10.3390/ma19214720)
Supplement: Supplementary file 1 [file materials-19-04720-s001.zip › materials-4622116-supplementary.pdf]

Supplementary Materials

Role of niobium and tantalum on the diffusivity of point defects in the passive film of TiZrNbTa multi-principal element alloys in sodium chloride solution

Zening Wang, Jinping Wu, Yi Zhang, Yu Yan * and Lijie Qiao

Beijing Advanced Innovation Center for Materials Genome Engineering, Corrosion and Protection Center, Institute for Advanced Materials and Technology, University of Science and Technology Beijing, Beijing 100083, China

* Correspondence: yanyu@ustb.edu.cn

1. Supplementary Tables

Table S1. Fitting parameters of the EIS measurements for the passive films formed on the $Ti_{45}Zr_{45}Ta_5Nb_5$ alloy at different potentials.

Impedance parameters	0 V_{SCE}	0.5 V_{SCE}	1.0 V_{SCE}	2.0 V_{SCE}	3.0 V_{SCE}
R_s (Ω cm²)	25.15 ± 2.35	26.56 ± 1.38	22.24 ± 0.25	27.25 ± 0.86	26.35 ± 2.96
Q_1 (×10⁻⁵ Ω⁻¹ cm⁻² sⁿ)	6.54 ± 0.25	8.32 ± 0.13	5.25 ± 0.58	3.12 ± 0.39	2.89 ± 0.67
n_1	0.86 ± 0.0168	0.87 ± 0.0075	0.83 ± 0.0105	0.86 ± 0.0082	0.81 ± 0.0142
R_1 (10⁵ Ω cm²)	0.15 ± 0.03	0.23 ± 0.05	0.52 ± 0.06	2.63 ± 0.08	1.95 ± 0.12
Q_2 (×10⁻⁵ Ω⁻¹ cm⁻² sⁿ)	1.21 ± 0.14	0.67 ± 0.07	0.72 ± 0.08	0.43 ± 0.03	0.30 ± 0.02
n_2	0.94 ± 0.0125	0.96 ± 0.0113	0.93 ± 0.0112	0.94 ± 0.0065	0.95 ± 0.0054
R_2 (10⁵ Ω cm²)	30.42 ± 1.25	42.21 ± 0.92	45.86 ± 0.59	50.32 ± 0.86	55.59 ± 0.25
d (nm)	4.91 ± 0.58	7.37 ± 0.79	9.10 ± 0.96	13.91 ± 0.85	17.83 ± 0.92
χ^2 (10⁻⁴)	2.12	5.63	3.56	2.12	4.56

Table S2. Fitting parameters of the EIS measurements for the passive films formed on the $Ti_{35}Zr_{35}Ta_{15}Nb_{15}$ alloy at different potentials.

Impedance parameters	0 V_{SCE}	0.5 V_{SCE}	1.0 V_{SCE}	2.0 V_{SCE}	3.0 V_{SCE}
R_s (Ω cm²)	22.80 ± 0.56	25.85 ± 2.05	23.96 ± 0.97	28.89 ± 1.35	24.36 ± 1.15
Q_1 (×10⁻⁵ Ω⁻¹ cm⁻² sⁿ)	2.85 ± 0.51	3.89 ± 0.22	5.52 ± 0.96	6.52 ± 1.02	3.56 ± 0.83
n_1	0.83 ± 0.0152	0.88 ± 0.0074	0.82 ± 0.0135	0.85 ± 0.0095	0.83 ± 0.0125
R_1 (10⁵ Ω cm²)	0.25 ± 0.08	0.58 ± 0.06	0.86 ± 0.02	1.59 ± 0.03	3.85 ± 0.11
Q_2 (×10⁻⁵ Ω⁻¹ cm⁻² sⁿ)	1.15 ± 0.12	0.60 ± 0.05	0.77 ± 0.07	0.46 ± 0.02	0.31 ± 0.01
n_2	0.95 ± 0.0128	0.97 ± 0.0092	0.92 ± 0.0131	0.93 ± 0.0066	0.95 ± 0.0075
R_2 (10⁵ Ω cm²)	33.15 ± 0.62	45.33 ± 1.05	50.89 ± 0.86	53.69 ± 0.31	58.38 ± 0.56
d (nm)	4.48 ± 0.46	7.13 ± 0.63	8.92 ± 0.85	13.53 ± 0.72	16.64 ± 0.63
χ^2 (10⁻⁴)	1.23	3.52	2.65	5.38	2.68

Table S3. Fitting parameters of the EIS measurements for the passive films formed on the $Ti_{30}Zr_{30}Ta_{20}Nb_{20}$ alloy at different potentials.

Impedance parameters	0 V_{SCE}	0.5 V_{SCE}	1.0 V_{SCE}	2.0 V_{SCE}	3.0 V_{SCE}
R_s (Ω cm^2)	25.10 ± 2.36	21.55 ± 0.88	22.58 ± 2.89	23.68 ± 0.08	24.55 ± 1.58
Q_1 (×10^{-5} Ω^{-1} cm^{-2} s^n)	3.52 ± 0.10	5.68 ± 0.46	5.14 ± 0.06	3.58 ± 0.82	8.27 ± 1.69
n_1	0.86 ± 0.0185	0.82 ± 0.0096	0.85 ± 0.0108	0.87 ± 0.0074	0.80 ± 0.0153
R_1 (10^5 Ω cm^2)	0.35 ± 0.08	0.29 ± 0.05	0.56 ± 0.03	3.86 ± 0.02	5.83 ± 0.02
Q_2 (×10^{-5} Ω^{-1} cm^{-2} s^n)	1.46 ± 0.17	0.84 ± 0.07	0.70 ± 0.04	0.52 ± 0.03	0.35 ± 0.01
n_2	0.93 ± 0.0126	0.95 ± 0.0092	0.96 ± 0.0081	0.92 ± 0.0060	0.94 ± 0.0076
R_2 (10^5 Ω cm^2)	38.15 ± 0.26	49.33 ± 0.51	52.89 ± 1.10	59.69 ± 0.84	62.38 ± 0.76
d (nm)	4.17 ± 0.50	6.02 ± 0.52	8.74 ± 0.68	12.74 ± 0.72	15.60 ± 0.65
χ^2 (10^{-4})	2.45	5.50	3.85	2.27	6.86

Table S4. Diffusion coefficients (D_0) of point defects in the passive film formed on the $Ti_{45}Zr_{45}Ta_5Nb_5$ alloy at different potentials.

E_{ff} (V_{SCE})	0	0.5	1.0	2.0	3.0
D_0 (×10^{-16} cm^2·s^{-1})	1.02 ± 0.29	2.59 ± 0.62	6.42 ± 1.43	15.43 ± 1.47	54.48 ± 3.21

Table S5. Diffusion coefficients (D_0) of point defects in the passive film formed on the $Ti_{35}Zr_{35}Ta_{15}Nb_{15}$ alloy at different potentials.

E_{ff} (V_{SCE})	0	0.5	1.0	2.0	3.0
D_0 (×10^{-16} cm^2·s^{-1})	0.38 ± 0.09	0.83 ± 0.25	1.31 ± 0.28	1.91 ± 0.45	3.20 ± 0.48

Table S6. Diffusion coefficients (D_0) of point defects in the passive film formed on the $Ti_{30}Zr_{30}Ta_{20}Nb_{20}$ alloy at different potentials.

E_{ff} (V_{SCE})	0	0.5	1.0	2.0	3.0
D_0 (×10^{-16} cm^2·s^{-1})	0.16 ± 0.11	0.31 ± 0.20	0.62 ± 0.19	0.94 ± 0.33	1.45 ± 0.35
